# Molecular chaperones in the acquisition of cancer cell chemoresistance with mutated *TP53* and MDM2 up-regulation

**DOI:** 10.18632/oncotarget.18899

**Published:** 2017-06-30

**Authors:** Zuzanna Tracz-Gaszewska, Marta Klimczak, Przemyslaw Biecek, Marcin Herok, Marcin Kosinski, Maciej B. Olszewski, Patrycja Czerwińska, Milena Wiech, Maciej Wiznerowicz, Alicja Zylicz, Maciej Zylicz, Bartosz Wawrzynow

**Affiliations:** ^1^ International Institute of Molecular and Cell Biology, Warsaw, Poland; ^2^ Institute of Biochemistry and Biophysics, PAS, Warsaw, Poland; ^3^ Postgraduate School of Molecular Medicine, Medical University of Warsaw, Warsaw, Poland; ^4^ Faculty of Mathematics, Informatics, and Mechanics, University of Warsaw, Warsaw, Poland; ^5^ Faculty of Mathematics and Information Science, Warsaw University of Technology, Warsaw, Poland; ^6^ Nencki Institute of Experimental Biology, PAS, Warsaw, Poland; ^7^ Laboratory of Gene Therapy, Department of Cancer Immunology, The Greater Poland Cancer Center, Poznan, Poland

**Keywords:** apoptosis, heat shock protein (HSP), mutant p53 gain-of-function, mouse double minute 2 homolog (MDM2), p73 tumor suppressor

## Abstract

Utilizing the TCGA PANCAN12 dataset we discovered that cancer patients with mutations in *TP53* tumor suppressor and overexpression of *MDM2* oncogene exhibited decreased survival post treatment. Interestingly, in the case of breast cancer patients, this phenomenon correlated with high expression level of several molecular chaperones belonging to the HSPA, DNAJB and HSPC families. To verify the hypothesis that such a genetic background may promote chaperone-mediated chemoresistance, we employed breast and lung cancer cell lines that constitutively overexpressed heat shock proteins and have shown that HSPA1A/HSP70 and DNAJB1/HSP40 facilitated the binding of mutated p53 to the TAp73α protein. This chaperone-mediated mutated p53–TAp73α complex induced chemoresistance to DNA damaging reagents, like Cisplatin, Doxorubicin, Etoposide or Camptothecin. Importantly, when the *MDM2* oncogene was overexpressed, heat shock proteins were displaced and a stable multiprotein complex comprising of mutated p53-TAp73α-MDM2 was formed, additionally amplifying cancer cells chemoresistance. Our findings demonstrate that molecular chaperones aid cancer cells in surviving the cytotoxic effect of chemotherapeutics and may have therapeutic implications.

## INTRODUCTION

Chemotherapy is a powerful tool aimed at cancer cell destruction. Nevertheless, in many cases, initially sensitive cancer cells rapidly develop acquired resistance. Chemoresistance in cancer cells is mediated by numerous mechanisms, which include drug inactivation, alteration of drug transporters that efflux anticancer drugs, enhanced DNA repair, tailored miRNA expression, epithelial-mesenchymal transition and activation of antiapoptotic and cell-survival pathways [1, 2].

The p53 tumor suppressor protein is activated during chemotherapy and plays a crucial role in apoptosis induction by DNA-damaging agents [3]. Wild type p53 (WT p53) is a flexible and conformationally labile protein, which under normal, non-stress conditions is efficiently ubiquitinated by MDM2 E3 ubiquitin ligase and, in consequence, targeted for proteasome mediated degradation [4]. Disruption of p53 function is often a prerequisite for tumor development and its progression. Mutations in the *TP53* gene are acutely common in cancer cells [5–9]. The majority of them are missense mutations resulting in a single amino acid substitution clustered in the DNA binding domain of the p53 protein. These p53 mutations can be divided into at least two classes: those which perturb the global conformation of the DNA binding domain (structural mutations), and those that affect DNA binding without affecting the conformational stability of the domain (contact mutations). Many p53 tumor-associated mutants (mut p53), apart from the canonical loss of tumor suppressor activity, gain new oncogenic functions (GOF), which contribute to regulation of cancer metabolism and malignant progression including increased tumorigenesis and metastasis [10–15]. Most clinical studies suggest that p53 alterations in the case of non-small cell lung carcinoma (NSCLC) carry a worse prognosis and may be relatively more resistant to chemotherapy and radiation [16], for review see [17]. Nevertheless, the overall impact of *TP53* mutations on the progression of NSCLC is still controversial and most likely depends on the stage of cancer development. It was suggested that mutations in *TP53,* which do not disrupt p53 protein structure and function, are an independent prognostic factor of shorter survival in advanced NSCLC [18]. Contrary to these findings, a recent study proposes no direct link between *TP53* mutations and overall NSCLC patient survival. Rather, it suggests that intratumor genetic heterogeneity may be an important factor in determining the role of *TP53* mutations on the prognosis of early stage NSCLC patients [19]. Other findings propose that the loss of transcriptional activity of LKB1 tumor suppressor protein, in the presence of mut p53, may promote tumor malignancy ensuing poor prognosis for lung carcinoma patients, thus suggesting a critical role of *TP53* mutations in cancer development [20].

In the case of breast cancer, the clinical relevance of *TP53* mutations is closely linked to the molecular subtypes of the disease [21, 22]. *TP53* mutations were associated with a worse outcome for Luminal B, HER2-enriched and Normal-like subtypes, whereas no significant effect was observed in Basal-like and Luminal A subtypes. Additionally a definite correlation between the type of the *TP53* mutation and patient survival could not be established. Although, a subset of patients bearing missense mutations in the region encoding the DNA binding domain was prone to poor clinical outcome [22]. On the cellular level, while no correlation was found between the type of *TP53* mutation and sensitivity to chemotherapeutics in some studies [23, 24], others have shown that the propensity of *TP53* mutants to induce chemotherapy resistance is mutant- and drug-dependent [25, 26].

Recent studies have shown that structural homologs of p53 containing the transactivation domain (TA): TAp73 and TAp63 are also activated by chemotherapy, leading to tumor cell death [27, 28]. Moreover, ectopic expression of TAp73α in lung cancer cells enhanced their sensitivity to cisplatin and elevated the apoptotic response, independently of p53 [29]. Drug resistance associated with high levels of mut p53 partly results in the inhibition of TAp73 and TAp63 transcriptional activity caused by the formation of mut p53-TAp73 and mut p53-TAp63 complexes, respectively [26, 27, 30–34].

Elevated levels of MDM2 protein are commonly observed in human cancers [35–41]. In the presence or absence of functional p53, tumor cells which express high level of MDM2, show high invasive potential [42]. In addition, *MDM2* gene amplification was shown to be an independent adverse prognosis marker for NSCLC patients [43]. Up-regulation of MDM2 protein in cancer cells is caused by *MDM2* gene amplification, elevated transcription, increased stability of *MDM2* mRNA, enhanced translation and through misregulated posttranslational modifications [44–47]. Elevated transcription of *MDM2* gene is directed not only by WT p53, but also by the TGFβ/SMAD2/3 and RAS/RAF/MEK/ERK oncogenic pathways [48]. Several SNPs were identified in *MDM2* genes, including 309 T > G in the *MDM2* promoter sequence, resulting in increased expression and associated with dramatic increase in cancer incident and time of onset [49]. Although overexpression of MDM2 should be observed in the case of WT p53, occurrence of mutated p53 and MDM2 overexpression are not mutually exclusive [37, 50, 51]. In addition, cancers bearing mut p53 may also overexpress MDM2 [52–55]. Whether these two oncogenic events cooperate with each other in establishing oncogenic phenotypes or are selected in a cell type- or tumor-specific manner remains to be established. Accumulation of these two oncoproteins in a certain tumor may alter the biochemical nature of the tumor, its growth characteristics as well as clinical outcome. Some studies have reported a worse prognosis for tumors carrying *TP53* mutation and overexpressing *MDM2* [56].

Molecular chaperones, including members of the HSPA, DNAJ and HSPC heat-shock families [57] were shown to be expressed at high levels in a wide range of tumors [58–61]. Elevated expression of HSPA1A/HSP70 (a member of the HSPA family, an alternative name of HSP70 will be used throughout the paper) was correlated with poor prognosis and resistance to therapy in many human cancers [62–67]. Additionally the involvement of HSP70 in numerous crucial steps of carcinogenesis, such as stabilization of oncogene(s), cell death, replicative senescence inhibition, induction of tumor angiogenesis, invasion, initiation and metastasis is well documented [67]. It must be stressed that HSP70 is not working alone in these reactions, for example in order to recognize the specific protein substrate(s) it requires co-chaperones expressed by the *DNAJ* family [68]. These not only attract HSP70 to the appropriate substrate but also activate its ATPase activity [69–71]. It was shown before that mut p53 functionally interacts with DNAJB1/HSP40 (a member of the DNAJ family, the alternative name of HSP40 will be used throughout the paper), HSPA1/HSP70 or HSPA8/HSC70 (members of the HSPA family) and HSP90A/HSP90 (member of the HSPC family, referred to as HSP90 in this paper) [72, 73]. Moreover, recombinant HSP40 was shown to act as the initiator for the *in vitro* loading of other chaperones onto p53 R175H protein and formation of the p53 R175H-HSP40-HSP70-HOP-HSP90 multichaperone complex [73]. Formation of such a complex was described to be crucial in stabilizing mutant p53 and increasing its half-life in cancer cells [73–75]. It should be noted that heat shock proteins not only functionally interact with mut p53 [72, 73] but also transiently interact with WT p53 [73, 76–79]. These bipolar modes of action are determined by gross misregulation of the HSF1 dependent transcriptional program occurring during tumorigenesis [80, 81] and in part by the nature of the substrates which are recognized by the specificity factors, numerous protein members of the DNAJ family [57, 70].

Herein we show, using clinical and genomic information from the TCGA data sets, that a subset of cancer patients with elevated expression of *MDM2* and alterations in *TP53* exhibited lower survival rate post treatment. More importantly, this phenomenon correlated with elevated levels of DNAJB1/HSP40 and other chaperones and co-chaperones. In search of a molecular mechanism of how the accumulation of these two key oncoproteins could stimulate the acquisition of chemoresistance, we show that heat shock proteins (DNAJB1/HSP40 and HSPA1A/HSP70), apart from stabilizing mut p53, are also involved in the mut p53-TAp73α interaction in breast and lung cancer cells. These events result in TAp73α sequestration and decreased TAp73α-mediated drug-induced apoptosis. In addition, we show that elevated levels of MDM2 displace those molecular chaperones in the mut p53-TAp73α complex, leading to the formation of a multiprotein complex containing structural mut p53, TAp73α and MDM2, which further augments cancer cell chemoresistance.

## RESULTS

### Altered expression profile of heat shock proteins (HSPs) coincides with lower survival rate of cancer patients with mutated *TP53* and overexpressed *MDM2*

The occurrence of *TP53* mutations (mut *TP53*) in patients was shown to confer a worse overall and breast cancer-specific survival [22]. In addition, the combined effect of *TP53* mutation, *TP53* loss of heterozygosity (LOH) and *MDM2* amplification on mortality was proposed to be additive [22].

**Figure 1:**
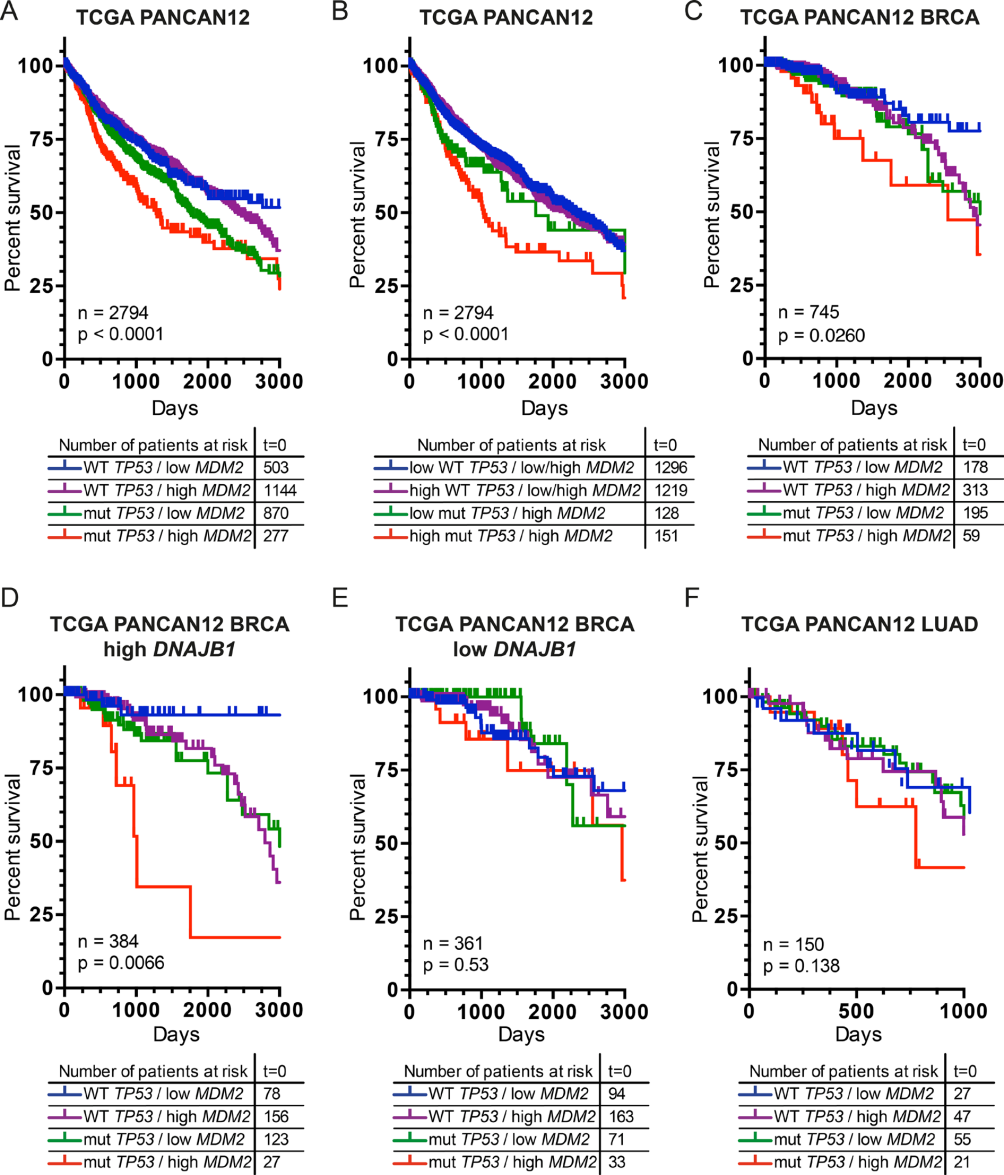
Cancer patients, with mutated *TP53* and simultaneous elevation of MDM2, exhibit decreased survival rate post treatment Kaplan-Meier survival curves depict percent survival of patients within the whole analyzed database or from particular cancer subtypes. (**A**) Cancer patients from the entire TCGA PANCAN12 dataset were analyzed with respect to their *TP53* mutation status and *MDM2* mRNA expression levels (*p =* 2.4e-06). (**B**) *TP53* mutation(s) linked with their expression levels and in addition with *MDM2* expression levels were considered for TCGA PANCAN12 patients. Expression of *TP53* gene, similar to *MDM2* was separated into low/high groups with the median expression used as a cutoff (*p =* 9e-05). (**C**) Breast Invasive Carcinoma (BRCA) patients with *TP53* mutation(s) and *MDM2* mRNA expression levels were analyzed (*p =* 0.026). Additionally, BRCA patients were divided into two cohorts depending on the level of *DNAJB1* gene expression, *DNAJB1* High (**D**) and *DNAJB1* Low (**E**). Subsequent analysis of *TP53* mutation status and *MDM2* mRNA expression levels followed independently for these two high/low groups with the median expression, derived from patients within the BRCA cohort, used as a cutoff (D) (*p =* 0.0066), (E) (*p =* 0.53). (**F**) Lung Adenocarcinoma (LUAD) patients with *TP53* mutation(s) and *MDM2* mRNA expression levels were analyzed (*p =* 0.138), median expression of *MDM2* derived from patients within the LUAD cohort. Statistical significance (*P* value) was verified by means of the log-rank test for trend.

To extend these findings, we utilized the TCGA PANCAN12 dataset obtained from Cancer Genomic Browser at UCSC [82]. Our initial global analysis focused on the survival rate of patients from all 12 distinct cancer subtypes present in the database with respect to their *TP53* status and *MDM2* expression level. The Kaplan-Meier survival curves indicate that the best prognosis for cancer patients associates with WT *TP53* and no overproduction of MDM2 (low *MDM2*) (Figure 1A). Mutation(s) in *TP53* alone (mut *TP53*), and to some extent *MDM2* overexpression (high *MDM2*) alone, resulted in the decrease of the survival rate. Interestingly, for roughly 10% of all cancer patients studied, when both events occurred simultaneously (mut *TP53*/high *MDM2*), the survival rate decreased significantly, especially in the early stages of cancer development (Figure 1A). Importantly, the lower survival rate of the (mut *TP53*/high *MDM2*) patients correlated with *TP53* loss of heterozygosity (LOH) (Supplementary Figure 1A), which suggests that the observed phenotype is due to gain-of-function (GOF) activity of mut *TP53*. Furthermore, when one considers *TP53* status in conjunction with its expression level and in addition with MDM2 elevation (Figure 1B), an even more profound decrease of the survival rate was observed (Figure 1B). Again, in this case *TP53* LOH was observed (Supplementary Figure 1B). This observation clearly strengthened the notion that the GOF activity of mut p53 is not only dependent on the nature of the somatic mutation in its gene but also on the level of expression and abundance within the cancer cell.

In the case of the Breast Invasive Carcinoma (BRCA) cohort from the TCGA PANCAN12 dataset, almost 8% of patients exhibit mutation(s) in *TP53* and overexpression of *MDM2* (59 cases). The Kaplan-Meier survival curves plotted for the BRCA patients once again indicate that the worst prognosis for breast cancer patients associates with simultaneous occurrence of *TP53* mutation(s) and *MDM2* overexpression (mut *TP53*/high *MDM2*) (Figure 1C). In terms of LOH, the second WT *TP53* allele is lost in 85% of the mut *TP53/*high *MDM2* cases (Supplementary Figure 1C). Patients from this group belong to: Basal, Luminal A&B breast cancer subtypes, suggesting a high degree of heterogeneity in comparison to the WT *TP53/*high *MDM2* patient cohort, where Luminal A subtype was predominant (Supplementary Figure 1D).

Interestingly, data mining experiments aimed at identifying potential correlations clearly indicate that the described phenomenon strongly corresponded to elevated level of heat shock protein DNAJB1/HSP40 (Figure 1D). In other words, from the BRCA cohort, patients with high expression of *DNAJB1/HSP40* exhibit poor survival rate dependent on *TP53* mutation(s) coupled with *MDM2* overexpression (mut *TP53/*high *MDM2*) (Figure 1D). For patients expressing low levels of *DNAJB1/HSP40* the correlation between poor prognosis and simultaneous presence of *TP53* mutation(s) coupled with elevated levels of MDM2 (mut *TP53/*high *MDM2* subgroup) is lost (Figure 1E). Therefore, one can assume that DNAJB1/HSP40, a well-characterized specificity factor for HSP70 responsible for attracting specific protein substrate(s), may be involved in the processes that decrease the survival of breast cancer mut *TP53/*high *MDM2* patients.

Poor survival rate of mut *TP53/*high *MDM2* breast cancer patients is not only correlated with the high expression level of DNAJB1/HSP40 molecular chaperone but also with high expression profile of other member of the DNAJ family, namely DNAJB6 [83] (Table 1). Interestingly, such a correlation was not observed for prominent members of the HSPA family, namely HSPA1A/HSP70 and HSPA8/HSC70, with the exception of HSPA6 and HSPA12B (Table 1). The HSPA6 protein is known to be a HSP40-independent molecular chaperone [84]. HSPA12B was shown to facilitate lung tumor growth [85]. Elevated expression of known oncogenic factor involved in breast cancer metastasis coding endoplasmic reticulum resident HSP90B1 (gp96, GRP94, Endoplasmin) correlated also with low survival rate of mut *TP53/*high *MDM2* breast cancer patients, indicating that ER folding and maturation mechanisms are involved in this process. In addition, it is worth noticing that the low survival rate of mut *TP53/*high *MDM2* breast cancer patients correlated with low level of constitutively expressed DNAJB4 and HSP90AB1/HSP90β (Table 1). This may suggest that high levels of these proteins can buffer the level of specific co-chaperones and chaperones, such as: DNAJB1, DNAJB6, HSPA6 in binding to proper protein substrates (Table 1).

**Table 1:** Correlation between expression of selected genes from the *HSP* superfamily with poor survival prediction of BRCA patients with mut *TP53* and high *MDM2*

Gene name	High level	Low level
*HSPA1A*	0.34	0.061
*HSPA1L*	0.018	**0.0083**
*HSPA2*	0.057	**0.011**
*HSPA5*	0.12	**0.044**
*HSPA6*	**0.0059**	0.24
*HSPA7*	0.023	0.43
*HSPA8*	0.05	0.48
*HSPA9*	0.8	**0.0046**
*HSPA12B*	**8.7e-06**	0.7
*HSPA13*	0.71	**0.018**
*HSPA14*	0.069	**1.2e-05**
*DNAJB1*	**0.0051**	0.19
*DNAJB4*	0.64	**6.7e-04**
*DNAJB5*	0.064	**0.034**
*DNAJB6*	**2.8e-04**	0.96
*DNAJB12*	0.27	**0.034**
*HSP90AA1*	**0.054**	0.33
*HSP90AB1*	0.94	**2.7e-05**
*HSP90B1*	**0.0081**	0.67

The table represents *P* values, obtained by means of log-rank test for trend. For each given gene the Breast Invasive Carcinoma (BRCA) cohort was dichotomized into two groups (High level/Low level), with the median expression of the given gene as the cutoff. Afterwards comparison of Kaplan-Meier survival curves of mut *TP53*/high *MDM2* patients vs rest in the groups was carried out. The correlations with statistical significance are highlighted in bold.

In the case of the Lung Adenocarcinoma (LUAD) cohort, within the TCGA PANCAN12 dataset, a very limited number of cases with information regarding *TP53* status and *MDM2* expression could be distinguished (*n =* 150). Nonetheless, patients who possess *TP53* mutation(s) and elevated levels of MDM2 at the same time (21 cases, 14%) exhibit a decreasing trend in the survival rate compared to others, yet devoid of statistical significance (Figure 1F). Thus, given the limited number of the LUAD cohort no further survival analysis was carried out.

The majority of patients in the breast cancer and lung cancer cohorts underwent at least one chemotherapy cycle. Unfortunately, primary and acquired resistance to chemotherapy is the major challenge in improving patient outcome in lung and breast cancer. In the case of NSCLC, resistance to Cisplatin and Doxorubicin was documented in 63 and 75 percent of patients, respectively [86]. Moreover, chemotherapy cycles in the case of breast cancer were proposed to be an accelerant leading to intratumoral heterogeneity (ITH) of the cancer and facilitating selection of cancer cells highly resistant to drug treatment [87]. Taking this into account, we hypothesized that patients who possessed both *TP53* mutations and overexpressed *MDM2* (mut *TP53*/high *MDM2*) acquired chemoresistance more efficiently, thus decreasing their chance of response to classical chemotherapy. Therefore, we set out to investigate on a cellular level, the interplay between *TP53* mutational status coupled with elevation of MDM2 and their combined effect on chemoresistance.

### Cancer cell chemoresistance is linked to p53 status, sequestration of TAp73α and type of chemotherapy used

Tumor derived “hot-spot” mutant variants of p53 were shown to have differential effects on the resistance of cultured cells to chemotherapy [25]. By utilizing two different cancer cell line models (breast – MCF7, SKBR3 and non-small cell lung cancer – H1299), we were able to show, in our control experiments, that cells expressing the conformational mutant (p53 R175H) and contact mutant (p53 R273H) developed chemoresistance to DNA damage chemotherapeutic treatment more efficiently than expressing the WT p53 (Supplementary Figures 2 and 3). In the case of NSCLC cell line (H1299) the development of the chemoresistance was more efficient for structural than for contact mutant expression (Supplementary Figure 3). Moreover, the results of experiments with SIMP peptides, which disrupt the p53 R175H-TAp73α complex [88], suggest that this complex may in part mediate the acquisition of chemoresistance by sequestrating TAp73α (Supplementary Figure 4A). The chemoresistance of cells expressing contact p53 R273H is not inhibited by SIMP peptides, suggesting that the nature of chemoresistance of cancer cells expressing p53 R175H and p53 R273H are different (Supplementary Figure 4C, 4D). Sequestration of TAp73α, which leads to inhibition of drug induced apoptosis, occurs only in the case of drugs inducing DNA double strand breaks (Cisplatin, Doxorubicin, Etoposide, Camptothecin) (Supplementary Figure 4). Interestingly, such an effect was not observed when cells were treated with Taxol, which is a cytoskeletal drug that acts as a mitotic inhibitor (Supplementary Figure 4E). Moreover, the presence of SIMP6 peptide did not increase the apoptosis (measured by the amount of PARP cleaved) in the presence of Taxol, suggesting that Taxol-dependent apoptosis of cancer cells is not driven by TAp73α (Supplementary Figure 4F).

The interpretation of the results regarding the expression of structural mutant (p53 R175H) in MCF7 is more complicated. Although cells stably expressing p53 R175H demonstrated higher proliferation post DOXO treatment than cells expressing p53 R273H (Supplementary Figure 2B–2D), the level of the structural mutant was higher than the contact one (Supplementary Figure 2A). The same observation concerns the inhibition of drug-induced apoptosis of cells expressing the appropriate variant of p53 (Supplementary Figure 2E–2G).

**Figure 2:**
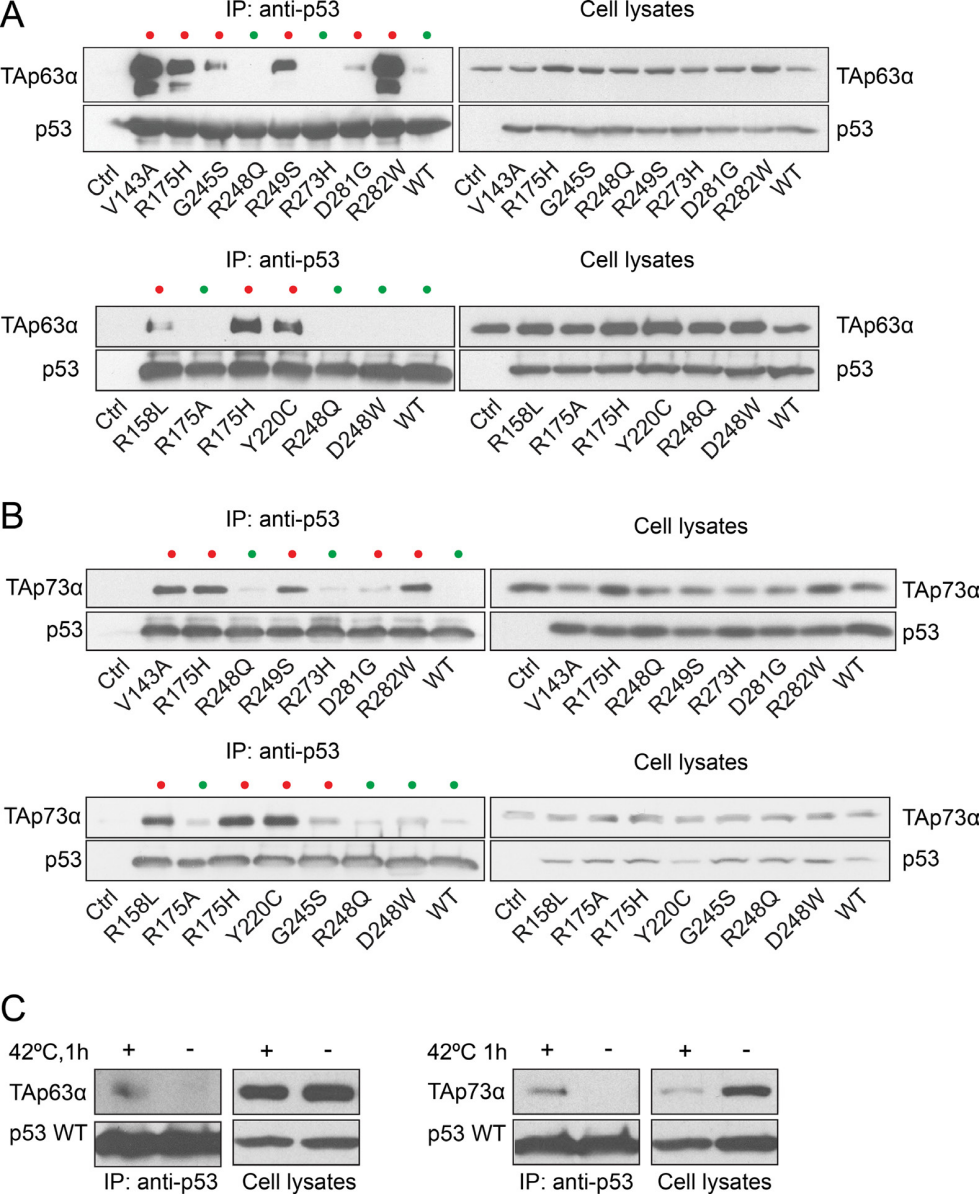
p53 mutants with conformational change form a stable complex with TAp63α and TAp73α Cells were transfected with plasmids encoding different p53 mutants or wild type (WT) p53 and TAp63α (**A**) or TAp73α (**B**). 24 h post-transfection cells were lysed and p53 protein was immunoprecipitated with anti-p53 antibody. The immunoprecipitated protein complexes were analyzed by Western blot. Cell lysates lacking p53 (Ctrl) were used as a control of the specificity of antibody used for immunoprecipitation. For clarity, conformational p53 mutants are highlighted with red dots and contact mutants with green dots. (**C**) Thermally unfolded p53 binds TAp63α and TAp73α isoforms. H1299 cells were transfected with plasmids encoding WT p53 and TAp63α or TAp73α. After 24 h cells were subjected to heat shock (42°C, 1 h), lysed and p53 protein was immunoprecipitated with anti-p53 antibody. The immunoprecipitated protein complexes were analyzed by Western blot.

The sequestration mechanism of TAp63 and TAp73 was shown to depend on the formation of a stable complex with a subset of tumor-derived mut p53s [26, 89–91]. To further investigate the binding affinity of TAp63 and TAp73 towards 11 different mutant forms of p53, we transfected p53-null human carcinoma H1299 cell line [92] with wild type (WT) or mut p53 and performed co-immunoprecipitation of p53 with ectopically expressed TAp63α or TAp73α (Figure 2A, 2B). Interestingly, WT p53 or mut p53 without global conformational changes (contact mutants) exhibited weak interaction with TAp63α and TAp73α (see lane WT p53, R248Q, D248W, R175A and R273H - green dots). However, mut p53 with altered global conformation (structural mutants: R175H, G245S, R249S, D281G, R282W, R158L, Y220C and V143A at non-permissive temperature –37°C - red dots) formed a stable complex both with TAp63α and TAp73α (Figure 2A, 2B).

In order to verify whether binding to mut p53 protein could effectively sequester the pro-apoptotic activity of TAp73α and TAp63α, we employed a transcriptional activation assay coupled with endogenous target transcript quantification. The *BAX* gene, being a pro-apoptotic target of TAp63 and TAp73 transcription factors [93], was investigated. The obtained data indicate that ectopic expression of p53 R175H protein inhibited TAp63α- or TAp73α-dependent reporter gene transcription from the *BAX* promoter in a dose dependent manner (Supplementary Figure 5A). In addition, endogenous *BAX* mRNA levels were efficiently decreased by expression of p53 R175H (Supplementary Figure 5B), in contrast to p53 R273H, which had little to no effect on the level of *BAX* mRNA (Supplementary Figure 5B). In effect, these results suggest that structural rearrangements of p53 are responsible for the sequestration of pro-apoptotic transcriptional activity of TAp63 and TAp73 tumor suppressor proteins.

To support these findings we showed that WT p53 protein expressed in cells at 37°C did not interact with TAp73α or TAp63α (Figure 2A, 2B). However, when the cells were exposed to heat shock (42°C, 1 h), a conformational shift within p53 occurred [75, 78] and increased the p53 binding to TAp63α or TAp73α (Figure 2C). It is known that partially unfolded, denatured proteins including conformational mutants of p53 or partially unfolded WT p53 protein are recognized by heat shock proteins [75, 78]. This suggests that heat shock proteins can influence the formation of TAp73α or TAp63α complexes with p53, which possessed altered conformation. Previously we have shown that overexpression of HSPA1A/HSP70 in cancer cells could dissociate, in ATP-dependent reaction, the p53 R175H-TAp63α but not p53 R175H-TAp73α complex [75]. Released TAp63α, via HSP70-dependent reaction, was able to initiate gene transcription from the *BAX* promoter. Contrary to TAp63α, the complex of TAp73α with p53 R175H was not dissociated in the presence of HSPA1A/HSP70 and the transcriptional activity of TAp73α remained inhibited [75]. In light of this, we sought to investigate the role of heat shock proteins in the formation of the p53 R175H-TAp73α complex in more detail.

### HSPs facilitate TAp73α and structural mutant p53 binding, thus increasing cancer cell chemoresistance

**Figure 3:**
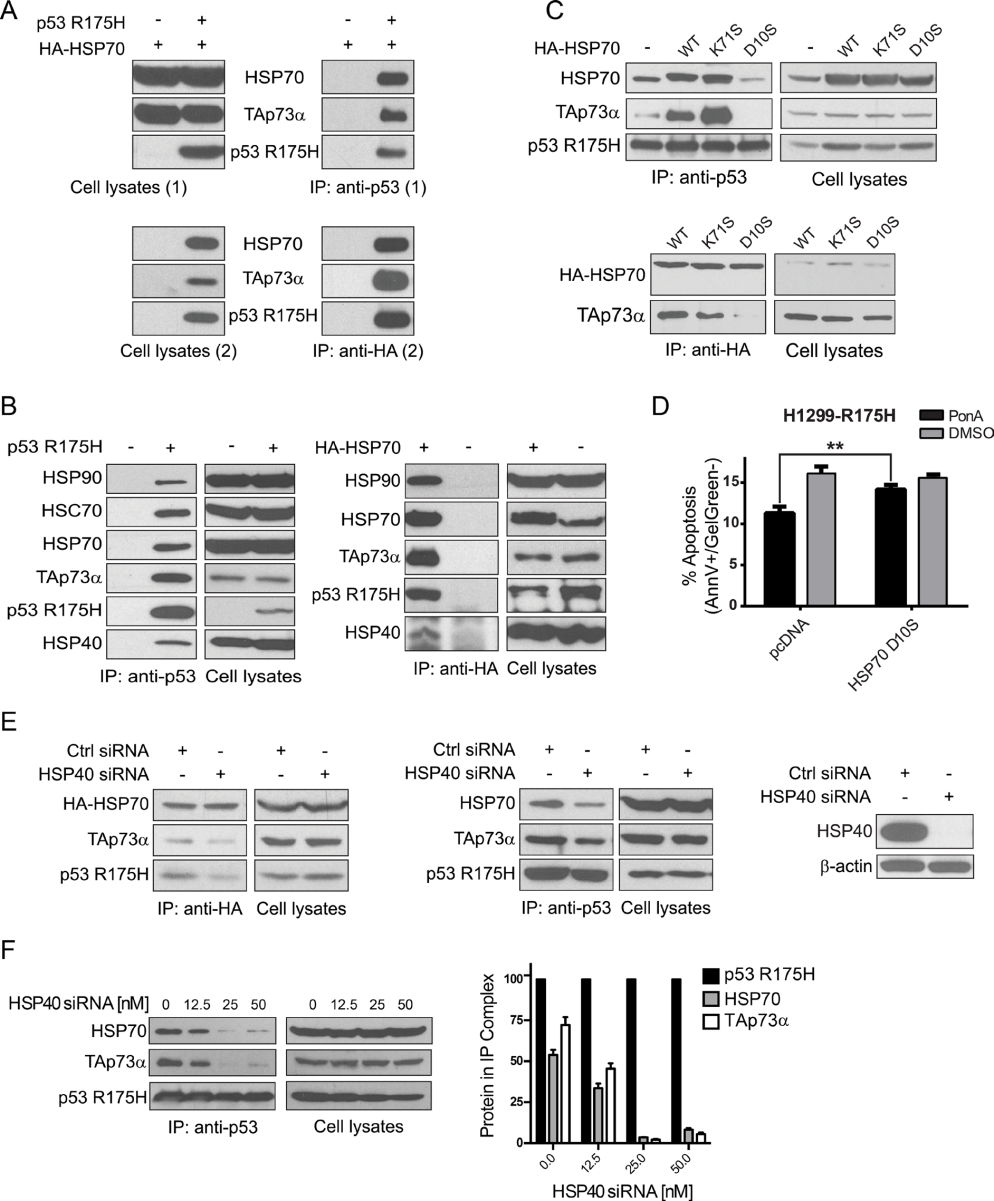
Molecular chaperones facilitate p53 R175H-TAp73α complex formation (**A**) Double immunoprecipitation experiments (Two step Co-IP). H1299 cells were transfected with plasmids encoding p53 R175H, TAp73α and HA-HSP70. After 24 h cellular proteins were cross-linked and the first co-immunoprecipitation (1) with anti-p53 antibody was carried out. Protein complexes were eluted from beads (Cell lysates 2) and second co-immunoprecipitation (2) with anti-HA antibody was performed. Laemmli buffer was supplemented with DTT to reverse cross-linking reaction. Lysates from cells transfected with plasmids encoding TAp73α and HA-HSP70 but not p53 R175H were used as a control of the specificity of anti-p53 antibody (first lane, right panel). (**B**) H1299 cells were transfected with plasmids encoding p53 R175H and TAp73α (left panel) or p53 R175H, TAp73α and HA-HSP70 (right panel). Immunoprecipitations were carried out with anti-p53 (left panel) or anti-HA antibody (right panel) to immunoprecipitate p53 or HA-HSP70, respectively. As a control of the specificity of antibodies applied for immunoprecipitations, cell lysates with no p53 R175H or no HA-HSP70 were used (–). (**C**) H1299 cells were transfected with plasmids encoding p53 R175H, TAp73α and HA-HSP70 WT/K71S/D10S respectively. 24 h post-transfection cells were lysed and p53 protein was immunoprecipitated with anti-p53 antibody – top panel, or with anti-HA antibody – bottom panel. The immunoprecipitated protein complexes were analyzed by Western blot. (**D**) H1299-R175H cells were transfected with a plasmid encoding HA-HSP70 D10S or with a control plasmid (pcDNA). After 6 h the medium was supplemented with 0.5 μM Ponasterone A (Pon A) to induce p53 R175H. Cells with no induction were treated with DMSO. After 24 h treatment with 60 μM Cisplatin, the apoptotic response of cells stained with Annexin V/Gel Green dye was measured by FACS. Bars represent the decrease (%) of cells in early apoptosis (Annexin V positive, Gel Green dye negative), normalized to non-treated control. Statistical significance (*P* value) was counted for three independent experiments with Anova statistical test. ** indicates statistical significance *p <* 0.01. (**E**) HSP40 molecular chaperone drives the formation of the complex p53 R175H-TAp73α with HSP70. H1299 cells were transfected with plasmids encoding p53 R175H, TAp73α and HA-HSP70 (left Western blot panel) or p53 R175H and TAp73α (middle panel). 18 h post the initial transfection, the cells were transfected once more with siRNA (50 nM) silencing the expression HSP40 (efficacy of silencing showed on the far right panel). Immunoprecipitations were carried out with anti-HA antibody (left Western blot panel) or anti-p53 antibody (middle panel) to immunoprecipitate HA-HSP70 or p53, respectively. (**F**) Dissociation of the p53 R175H-TAp73α complex is caused by HSP40 inhibition in a dose dependent manner. The procedure was carried out analogously to (E) with the secondary transfection comprising a range of HSP40 siRNA (0–50 nM) in two-fold dilutions, as depicted. Immunoprecipitations were carried out with anti-p53 antibody. Differences in the levels of bound HSP70 and TAp73α bound to p53 R175H were determined by densitometric analysis (right panel). The values obtained from three independent biological experiments were normalized to the level of immunoprecipitated p53 R175H for each concentration of HSP40 siRNA used.

Consecutive double immunoprecipitation experiments (Two-step Co-IP) suggested that HSPA1A/HSP70 could interact with p53 R175H and TAp73α simultaneously (Figure 3A). Apart from HSP70, endogenous molecular chaperones DNAJB1/HSP40 and HSP90A/HSP90 co-immunoprecipitated with the protein complex p53 R175H-TAp73α (Figure 3B). We also detected that HSPA8/HSC70 bound to the p53 R175H-TAp73α complex (Figure 3B). In order to further investigate the role of HSPA1A/HSP70 in p53 R175H-TAp73α complex formation we used three forms of HSPA1A/HSP70 representing different states of nucleotide bound to the heat shock protein, namely HSP70 WT, HSP70 K71S and HSP70 D10S. HSP70 WT possesses ATPase activity [94]. The HSP70 K71S variant has abrogated ATPase activity, thus being mainly in an ADP-bound form, whereas HSP70 D10S possesses very low affinity to neither ATP nor ADP, thus not presenting any ATPase activity [95]. It has been shown before that ADP stabilized substrate-HSP70 complex formation [96]. Both ATPase-dead variants demonstrate a dominant negative mode towards HSP70 WT [95]. Overexpression of the three HSP70 variants in H1299 cells (ectopic expression of HA-HSP70 WT, K71S and D10S, respectively) resulted in the stabilization of the p53 R175H-TAp73α complex for HSP70 WT and even stronger for K71S, but induced the destabilization of the complex for the HSP70 D10S variant (Figure 3C upper panel). In addition, the interaction of TAp73α with HSP70 variants was investigated (Figure 3C lower panel). HSP70 WT and HSP70 in the ADP bound form (HSP70 K71S) interacted with TAp73α, but in the case of HSP70 nucleotide free form (HSP70 D10S) the interaction with TAp73α was abrogated. Furthermore, overexpression of HSP70 D10S restrained p53 R175H-dependent inhibition of apoptosis in our H1299 cell model, where expression of p53 R175H was induced by PonA (Figure 3D, see also Supplementary Figure 3A for controls), thus strongly suggesting that HSPA1A/HSP70 is actively involved in the p53 R175H-TAp73α complex formation.

As shown, the survival rate of cancer patients who possess mut p53 in concert with MDM2 elevation (mut *TP53/*high *MDM2*) decreased substantially for the group of patients with elevated DNAJB1/HSP40 levels (Figure 1D). Hence, this molecular chaperone could be involved in the formation of specific protein-protein complexes resulting in the sequestration of TAp73α tumor suppressor. In support of this hypothesis, endogenous DNAJB1/HSP40 knockdown by specific siRNA was carried out and resulted in partial dissociation of the p53 R175H-TAp73α complex (Figure 3E, 3F).

In summary, these results highlight the importance of molecular chaperones, namely HSPA1A/HSP70 and DNAJB1/HSP40 in facilitating the formation of the p53 R175H-TAp73α complex. Moreover, several other molecular chaperones like HSP90A/HSP90 or HSPA8/HSC70 also immunoprecipitated with this complex (Figure 3B) suggesting the existence of chaperone networks involved in sequestration of TAp73α, thus allowing cancer cells to survive in the presence of chemotherapeutics.

### Elevated levels of MDM2 release chaperones from mutant p53-TAp73α complex and induce the formation of a three-body mut p53-TAp73α-MDM2 complex, which augments cancer cell chemoresistance

Breast and lung cancer patients with elevated expression of *MDM2* in concert with alterations in *TP53* (mut *TP53/*high *MDM2*) exhibit lower survival rate post chemotherapy with respect to patients that possessed mutations in *TP53* or overexpressed *MDM2* independently (Figure 1). Having in mind the active role of HSP70-HSP40 chaperone machine in the p53 R175H-TAp73α complex formation, we investigated the potential effects of MDM2 cellular influx on TAp73α sequestration and the role of molecular chaperones in this process.

**Figure 4:**
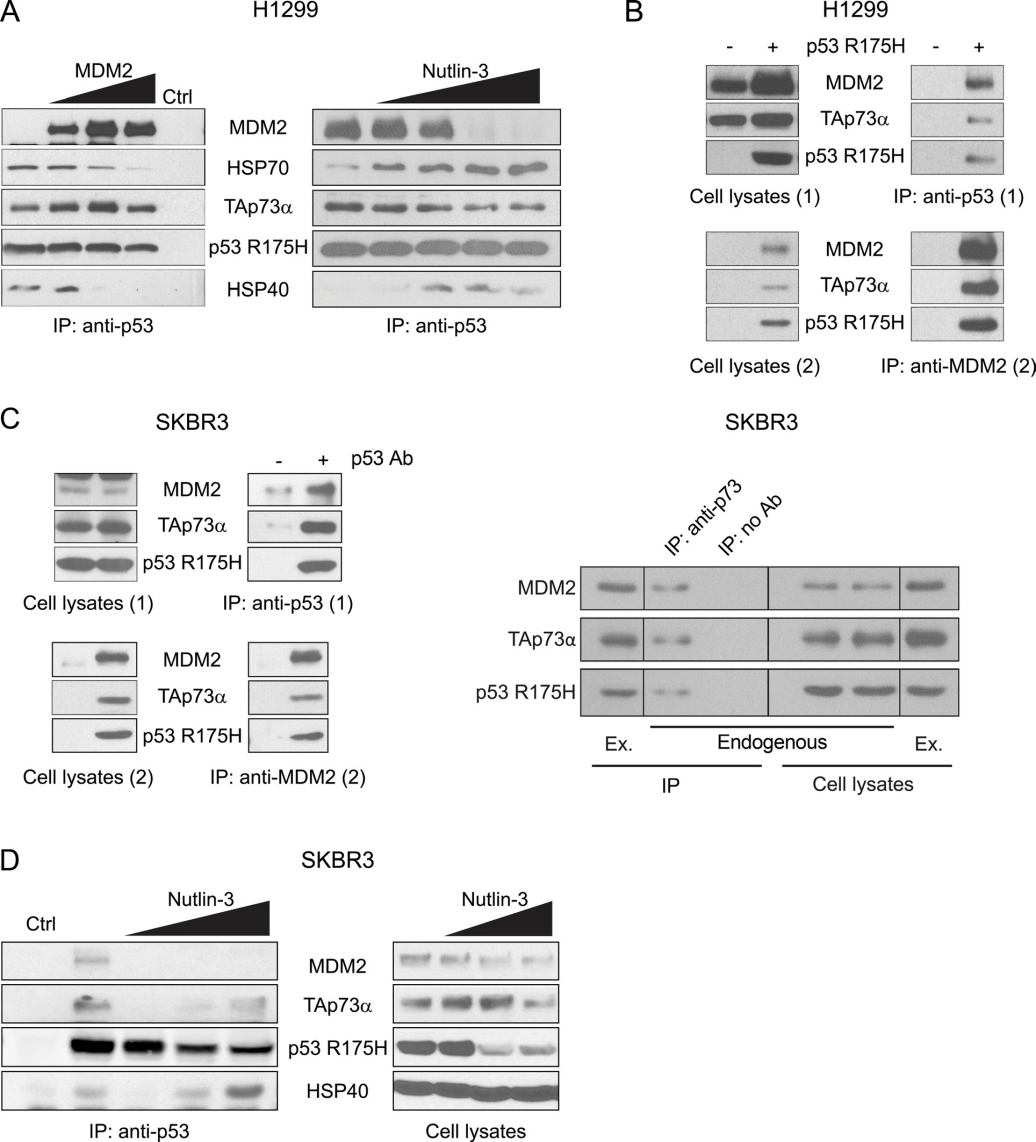
MDM2 dissociates HSP70 and HSP40 from the p53 R175H-TAp73α subcomplex and forms a three-body complex with p53 R175H and TAp73α (**A**) H1299 cells were transfected with plasmids encoding p53 R175H, TAp73α and MDM2, as indicated. 24 h post-transfection cells were lysed, protein complexes were immunoprecipitated with anti-p53 antibody and subjected to Western blot analysis. Lysates from cells lacking p53 R175H were used as a control of antibody specificity used for immunoprecipitation (Ctrl). (**B**) Double co-immunoprecipitation experiments (Two step Co-IP). H1299 cells were transfected with plasmids encoding p53 R175H, TAp73α and MDM2. After 24 h cellular proteins were cross-linked and first co-immunoprecipitation (1) with anti-p53 antibody was carried out. Protein complexes were eluted from beads (Cell lysates 2) and second co-immunoprecipitation (2) with anti-MDM2 antibody was performed. Laemmli buffer was supplemented with DTT to reverse cross-linking reaction. Lysates from cells transfected with plasmids encoding TAp73α and MDM2 but not p53 R175H were used as a control of the specificity of anti-p53 antibody. (**C**) Two step Co-IP with endogenous p53 R175H and MDM2 in SKBR3 cell line. Cells were transfected with a plasmid encoding TAp73α and the procedure was carried out as in (B) (left panel). Endogenous three-body complexes formed by p53 R175H, TAp73α and MDM2 were immunoprecipitated from SKBR3 cell lysates with anti-p73 antibody (right panel). (**D**) SKBR3 cell were treated with increasing concentrations of Nutlin-3 (0, 5, 10, 20 μM). After 24 h cellular proteins were cross-linked using 0.25 mM DSP and co-immunoprecipitation with anti-p53 antibody was carried out.

In the case of H1299 lung cancer cells, ectopic expression of MDM2 released HSPA1A/HSP70 and DNAJB1/HSP40 from the p53 R175H-TAp73α complex in a dose dependent manner, thus leading to the formation of p53 R175H-TAp73α-MDM2 complex (Figure 4A). Moreover, in the case of MDM2 overproduction, titration of Nutlin-3, a known MDM2 inhibitor [97, 98], reversed the reaction: MDM2 was released from p53 R175H-TAp73α complex and endogenous HSPA1A/HSP70 and DNAJB1/HSP40 rebound to the p53 R175H-TAp73α complex (Figure 4A). To support the notion that MDM2 simultaneously interacts with mut p53 and TAp73α, the two-step Co-IP methodology was employed. It revealed that under conditions where MDM2 was elevated, a stable three-body complex comprising of p53 R175H-TAp73α-MDM2 had formed (Figure 4B). Moreover, a similar complex was observed in the breast cancer SKBR3 cell line, which expresses endogenous p53 R175H (Figure 4C left panel). In control experiments we showed that endogenous p53 R175H simultaneously interacted with endogenous TAp73α and MDM2 forming a three-body complex (Figure 4C right panel). The pre-existing p53 R175H-TAp73α-MDM2 complex, composed from endogenous proteins, was also sensitive to increasing concentration of Nutlin-3 (Figure 4D). At low concentration of Nutlin-3 almost no detectable level of MDM2 and TAp73α was observed in the complex. However, further increase in Nutlin-3 concentration resulted in the appearance of HSP40 and TAp73α in the complex with p53 R175H. These results suggest that the presence of Nutlin-3 shifts the equilibrium from a three-body p53 R175H-TAp73α-MDM2 complex to a binary R175H-TAp73α structure and its formation is stimulated by the presence of molecular chaperones (Figure 4D left panel). Noticeably, in the appropriate input lanes (cell lysates, Figure 4D right panel) increasing concentration of Nutlin-3 decreased the total amount of p53 R175H and TAp73α. Nutlin-3 was shown before to decrease the stability of p53 R175H by destroying p53 R175H-multichaperone complex [74, 75]. Interestingly, similarly to the stability decrease of mut p53 in the presence of Nutlin-3, one can observe that the abundance of TAp73α was also affected. This makes the interpretation of the IP results more difficult, but could also open a new avenue of investigation of the role of molecular chaperones in stability of TAp73α and/or subsequent conversion of TAp73α to the different p73 isoforms.

**Figure 5:**
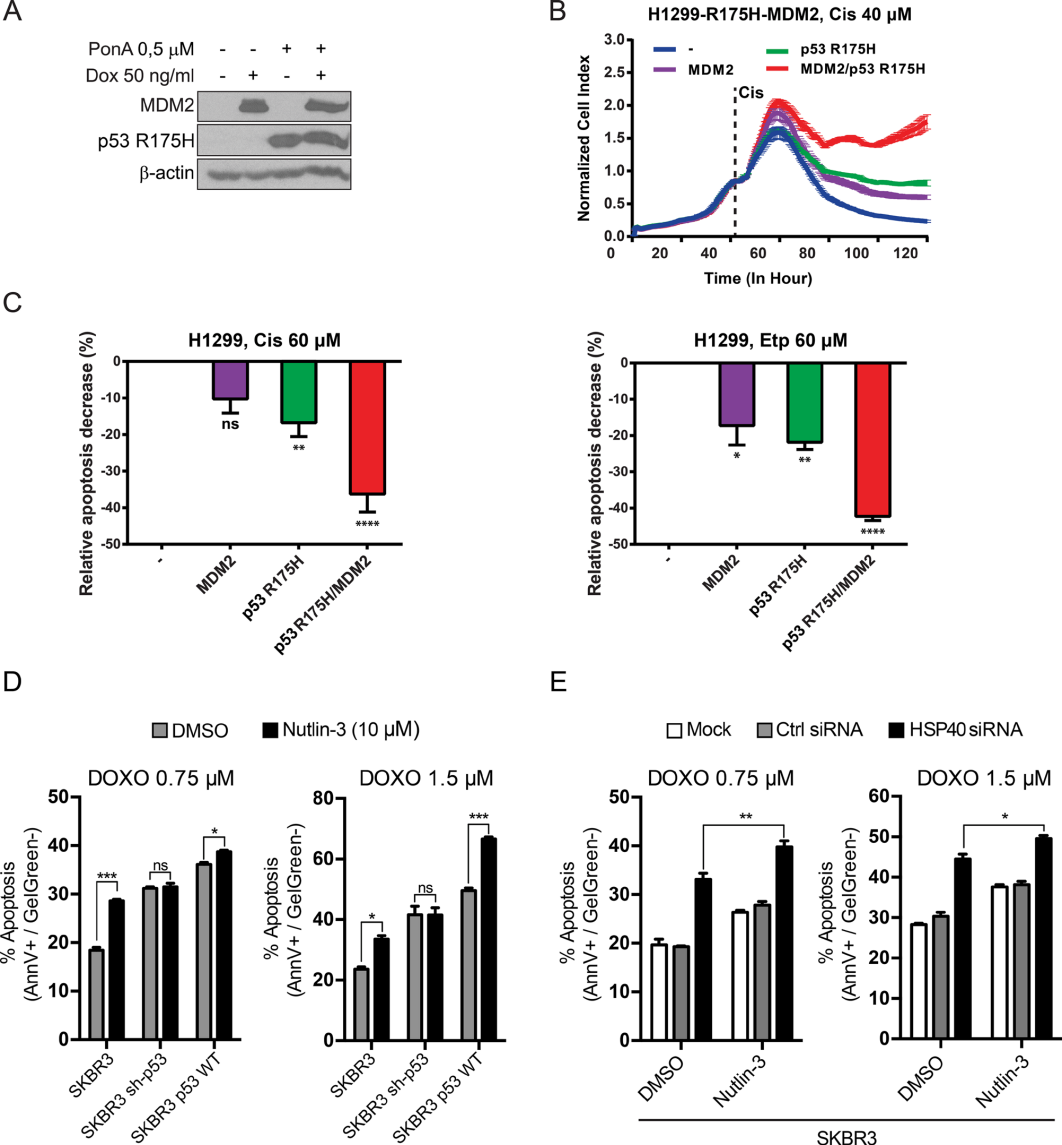
p53 R175H and MDM2 proteins synergistically reduce chemosensitivity of lung and breast cancer cells (**A**) H1299-R175H-MDM2 cell line was treated with Ponasterone A (Pon A) and/or Doxycycline (Dox) for 24 h to induce p53 R175H and/or MDM2, respectively. Immunoblotting with specific antibody revealed tight and efficient expression of both proteins. (**B**) Induced and uninduced cells were grown in triplicate in chambers compatible with the xCELLigence RTCA DP Instrument and Cisplatin (40 μM) was added at the indicated time point. Proliferative index was monitored for 120 h. Mean and standard deviation of three repeats are shown. (**C**) After 48 h treatment with 60 μM Cisplatin (left panel) or Etoposide (right panel), the apoptotic response of induced or uninduced cells stained with Annexin V/Gel Green dye was measured with a flow cytometer. p53 R175H or MDM2 expressed alone reduced apoptosis to same extent, whereas significant decrease was observed after simultaneous induction of both proteins. Bars represent the relative decrease (%) of cells in early apoptosis (Annexin V positive, Gel Green dye negative), estimated as follows $=\frac{\left(\text{Value}-\text{Baseline}\right)}{\text{Baseline}}\times 100$ (Baseline–Apoptotic response of uninduced H1299-R175H-MDM2 cell line). Statistical significance (*P* value) was counted for three independent experiments with Anova statistical test. *, **, ***, **** indicate statistical significance *p <* 0.05, *p <* 0.01, *p <* 0.001, *p <* 0.0001, respectively. (**D**) Apoptotic response measurements of the SKBR3 derived cell lines treated with Doxorubicin (DOXO). The cells were exposed to 0.75 μM and 1.5 μM concentrations of DOXO. Nutlin-3 at 10 μM concentration was added to the cells at the same time as DOXO, DMSO was used as a negative control. An additional control of Nutlin-3 only treated cells was also carried out. 24–30 h post treatment the measurement of apoptotic cells was performed with flow cytometry. Bars represent the percentage of cells in early apoptosis (Annexin V positive, GelGreen negative), normalized to non-treated control. Statistical significance (*P* value) was counted for three independent experiments with Anova statistical test. (**E**) Apoptotic response measurements of the SKBR3 cells with HSP40 siRNA knockdown treated with Nutlin-3 and doxorubicin (DOXO). The cells were initially transfected at low density with HSP40 siRNA/Ctrl siRNA (50 nM) twice in 36-hour intervals (The efficacy of silencing was determined by WB analysis–data no shown). Afterwards the cells were exposed to DOXO and Nutlin-3 and the experiment was carried out analogously to (D). Statistical significance (*P* value) was counted for three independent experiments with Anova statistical test.

In order to elucidate the molecular mechanism leading to the sequestration of TAp73α tumor suppressor activity in the presence of mutant p53 and elevated levels of MDM2, we constructed a stable cell line (H1299) with inducible expression of MDM2 and/or p53 R175H (Figure 5A). Real-time proliferative index of these cells, in response to chemotherapeutic treatment, was measured with the xCELLigence system (Figure 5B). Exposure to Cisplatin revealed that cells, which were induced to express only the structural mutant p53 R175H, were more resistant to Cisplatin than the control, uninduced cells. Additionally, overexpression of MDM2 alone facilitated some chemoresistance elevation. Nevertheless, in the case when both p53 R175H and MDM2 were expressed simultaneously, the chemoresistance to Cisplatin was dominant (Figure 5B) which suggests a synergistic mode of action of the two proteins in question. Consistent with the described cytotoxic assays, induction of MDM2 or p53 R175H alone inhibited Etoposide/Cisplatin induced apoptosis of cancer cells, to some extent (Figure 5C). These effects were substantially increased when both, MDM2 and p53 R175H were overexpressed. This data suggests that the formation of p53 R175H-TAp73α-MDM2 complex could additionally decrease the cellular apoptotic response, thus increasing chemoresistance (Figure 5C). Interestingly, the presence of SIMP6 peptide, which was shown to inhibit p53 R175H-TAp73α complex formation (Supplementary Figure 4A), was not able to liberate TAp73α from the p53 R175H-TAp73α-MDM2 complex (Supplementary Figure 6). Thus, one could argue that the conformation of the proteins forming the p53 R175H-TAp73α complex is different than the conformation of the components forming the three-body p53 R175H-TAp73α-MDM2 complex. On the other hand, MDM2 can also act as a scaffold stabilizer due to the high affinity of this oncoprotein to p53 R175H and TAp73α within the three-body complex.

In the case of endogenous p53 R175H-TAp73α-MDM2 complex formed within the SKBR3 cell line, inhibition of MDM2 by Nutlin-3, which was shown to dissociate MDM2 from the three-body complex (Figure 4D), manifested a statistically significant increase in DOXO-induced apoptosis (Figure 5D). Thus, by inhibiting MDM2 it may be possible to partially rescue endogenous pro-apoptotic activity of the TAp73α tumor suppressor protein. In control experiments we showed that Nutlin-3 does not have any effect on apoptosis of SKBR3 sh-p53 cells, in which expression of endogenous p53 R175H was efficiently knocked down (Figure 5D). In addition, the DOXO induced apoptosis profile of SKBR3 p53 WT cells (stable knockdown of p53 R175H, constitutive expression of p53 WT) reveals that in this scenario Nutlin-3 acts as a canonical catalyst of the p53 response pathway, augmenting MDM2 trans-repression (Figure 5D). The simultaneous presence of Nutlin-3 and inhibition of HSP40 by specific siRNA additionally stimulated DOXO-induced apoptosis (Figure 5E). This suggests that in the SKBR3 background, similarly to H1299, formation of multiprotein complexes composed of p53 R175H, MDM2 efficiently sequesters TAp73α. Furthermore, simultaneous presence of MDM2 inhibitor and inhibitors of molecular chaperones can stimulate partial dissociation of these multiprotein complexes allowing drug dependent apoptosis of cancer cells.

## DISCUSSION

Molecular chaperones, co-chaperones, adaptors and folding enzymes are well known to be the cornerstones of dynamic multi-protein complexes, which regulate protein homeostasis including protein maturation and protein degradation (for review see [99–102]). A thorough comparative analysis of a large set of tumor specimens has shown, that under stress conditions such as malignant transformation, accelerated by MYC transcriptional activity, the chaperone network is extensively reshaped leading to the formation of stable multiprotein complexes (called the epichaperome) facilitating tumor survival, irrespective of tissue of origin or genetic background. Members of the HSPA and HSPC and co-chaperones of DNAJB families are the nucleating seeds for these physically and functionally integrated complexes present in over half of the all cancers tested [103].

Herein, using breast and lung cancer cell lines, we showed that molecular chaperones like DNAJB1/HSP40, HSPA1A/HSP70, and HSP90A/HSP90 formed a multiprotein complex that catalyzed the binding of TAp73α to mut p53. Formation of such a complex increased cancer cell survival in the presence of chemotherapeutics like Cisplatin, Doxorubicin, Etoposide or Camptothecin by inhibition of TAp73α-dependent apoptosis. The emergence of that pro-survival complex was dependent on the conformation of mutated p53 and the presence of molecular chaperones. Provided that the global conformation of p53 protein was changed (structural mutations or heat-shock exposure), mut p53-TAp73α immunoprecipitated with molecular chaperones and co-chaperones. Inhibition of HSP70 chaperone (by coexpression of dominant negative mutant HSP70 D10S) or inhibition of expression of HSP40 co-chaperone (by specific siRNA) suppressed the mut p53-TAp73α complex formation, which resulted in the increase of drug-dependent apoptosis.

**Figure 6:**
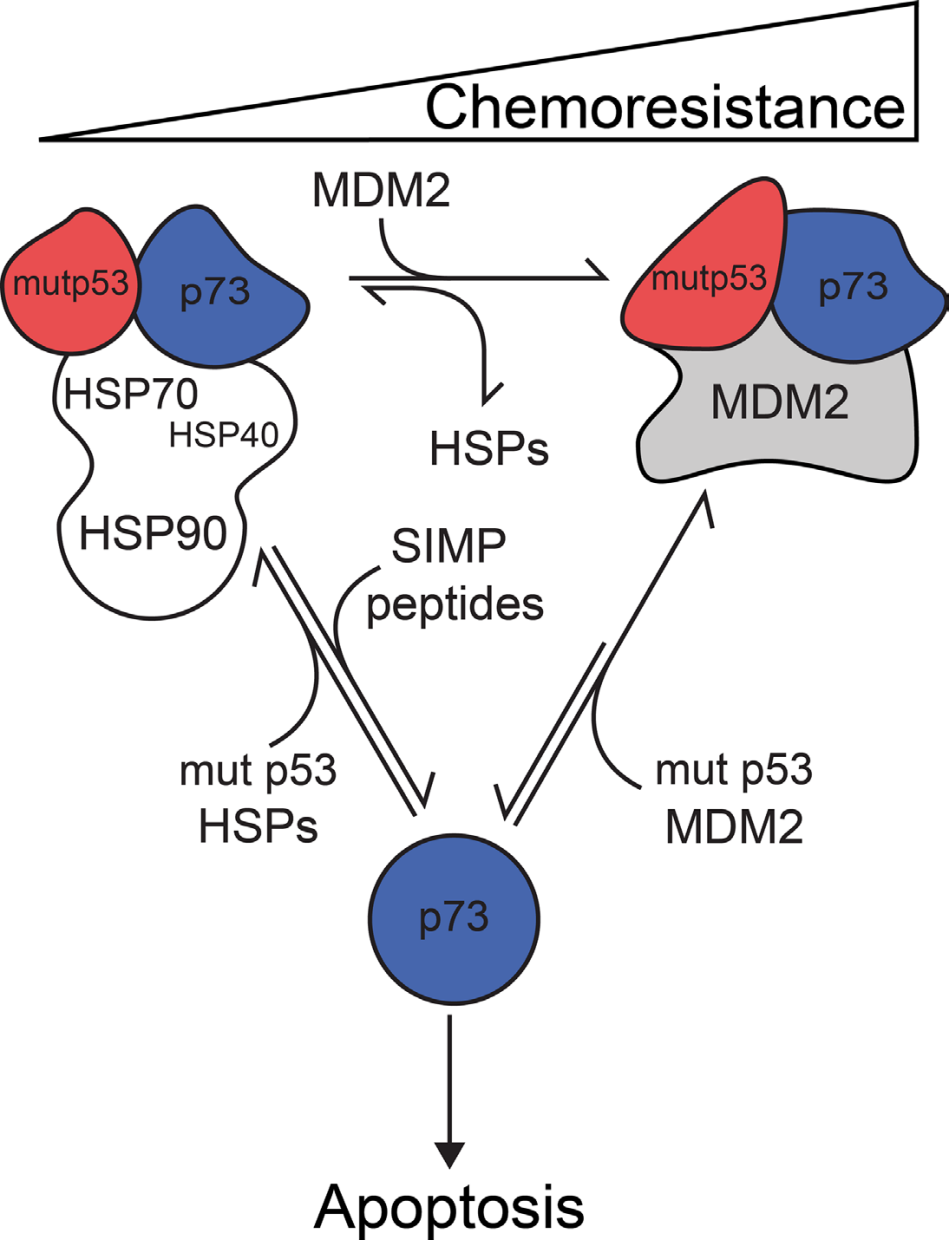
The structural complex comprising of mutant p53-TAp73-MDM2 implies a novel model of cancer cell chemoresistance The proposed model elucidates chaperone-mediated chemoresistance increase of cancer cells expressing mutant p53 and MDM2. In cancer cell, where endogenous levels of molecular chaperones (HSPs) are elevated, the interaction equilibrium between p53 structural mutant and TAp73α is shifted towards the formation of mut p53-TAp73α subcomplex. Formation of this complex inhibits TAp73α-dependent apoptosis, which results in cancer cell chemoresistance to DNA damage inducing drugs. Specific inhibitors like SIMP peptides oppose the mentioned equilibrium shift, thus increasing TAp73α-dependent apoptosis, which partially reduces cancer cell chemoresistance. MDM2 protein elevation displaces molecular chaperones from the complex and further shifts the equilibrium towards nuclear formation of the mut p53- TAp73α-MDM2 complex. However, in the situation when the initial levels of MDM2 are already elevated, we cannot exclude that the multi-protein complex is formed without the participation of molecular chaperones. Sequestration of TAp73α in this relatively stable complex significantly inhibits TAp73α-dependent apoptosis and intensifies cancer cell chemoresistance. The pro-oncogenic activities of mutant p53 can be manifested through other, non-sequestration based mechanisms [116]. We cannot exclude the possibility that other molecular chaperones and co-chaperones are also involved in these processes.

In addition, we have also shown that the chaperone network, which assists cancer cells to survive in the presence of chemotherapeutics, was completely remodeled after transformation with MDM2 oncoprotein. Molecular chaperone HSPA1A/HSP70 and co-chaperone DNAJB1/HSP40 dissociated and a stable multiprotein complex containing p53 R175H, TAp73α and MDM2 formed, additionally amplifying cancer cells chemoresistance. Moreover, the presented data suggests that MDM2-dependent reprograming of interactions between mut p53 and TAp73α requires an intermediate step in which molecular chaperones and co-chaperones are involved, albeit we cannot exclude the possibility that in some cancer cells this complex can be formed without the chaperone dependent intermediate (Figure 6).

In order to link our findings to clinical relevance, we showed that mutations in *TP53*, as well as loss of *TP53* heterozygosity coinciding with MDM2 up-regulation correlated with decreased survival for a subset of cancer patients within the TCGA PANCAN12 dataset. Importantly, this effect was due to the *TP53* loss of heterozygosity (LOH). If lower survival rate of the mut *TP53/*high *MDM2* patients was not accompanied by LOH of the second *TP53* allele, decreased survival rate for these patients could be interpreted by means of canonical MDM2 inhibition of WT p53 tumor suppressor activity. Careful analysis of the cancer subtypes within the PANCAN12 dataset indicated that this particular correlation manifests itself foremost in breast cancer (visible also for lung, ovarian and head and neck cancers – this paper and unpublished results).

Decreased survival of breast cancer patients with mut p53 and MDM2 elevation (mut *TP53/*high *MDM2*) strongly correlated with elevated levels of co-chaperone DNAJB1/HSP40 and selectively with other chaperones (Table 1). Survival rate analysis focused on the DNAJ family revealed that decreased survival of mut *TP53/*high *MDM2* breast cancer patients, not only correlated with high expression of *DNAJB1*, but also with high level of expression of *DNAJB6.* The latter has been shown to be associated with various diseases and physiological processes, like neurodegenerative diseases, infection diseases, limb-girdle muscular dystrophy, cardiomyocyte hypertrophy and cancer [83].

Interestingly, increased levels of the major heat inducible HSPA1A/HSP70 protein (highly overexpressed in cancer cells), and its constitutively expressed HSPA8/HSC70 counterpart, did not correlate with poor prognosis for mut *TP53/*high *MDM2* breast cancer patients. Most probably this phenomenon is due to the redundancy of substrate specificity for those molecular chaperones, which are directed by different DNAJ specificity factors. In addition, compensation effects resulting from differential gene expression within the *HSPA* family should not be ruled out [104, 105].

The significance of co-chaperone specificity factors, in recognition of particular protein substrates, was highlighted by HSPA6. Its high level of expression correlated with low survival of mut *TP53/*high *MDM2* breast cancer patients. We have previously shown that HSPA6 (its expression is induced in cancer) is a DNAJ-independent HSPA protein, which can reactivate heat-unfolded p53 without any DNAJ specificity factor [84].

High expression levels of endoplasmic reticulum resident HSP90B1 were shown to be associated with breast cancer metastasis [106] and cancer cell migration [107]. Hence, the observed correlation between elevated levels of HSP90B1 and poor survival rate mut *TP53/*high *MDM2* patients, might suggest that this particular subgroup is more susceptible to metastasis.

In summary, the expression levels of several molecular chaperones and co-chaperones were shown to correlate with decreased survival rate of mut *TP53/*high *MDM2* breast cancer patients (Table 1). These factors are likely to form functional network(s) that can help cancer cells survive the toxicity of chemotherapy. Moreover, remodeling of these networks, after MDM2 oncogene up-regulation, not only caused the release of HSP70 and HSP40 from the mut p53-TAp73α complex but also could initiate binding of other chaperones to mut p53-TAp73α-MDM2 complex. The presence of HSP70 with its co-chaperone HSP40 allowed for the formation of mut p53-TAp73α complex yielding increased cellular survival in the presence of chemotherapeutics. Chaperones in this case reveal their pro-survival and oncogenic nature. Nevertheless, we have previously shown that overexpression of HSP70 is required for ATP-dependent dissociation of the mut p53 R175H-TAp63α complex [75]. In that case the same molecular chaperones exhibited tumor suppressor activity.

Recently, a publication by Stindt et al. has shown that binding of mutant p53 to TAp63α and TAp73α is differentially modulated by MDM2 [108]. Moreover, the formation of p53 R175H-TAp73α-MDM2 complex was suggested in HCT116 (*TP53*^−/−^) cells with ectopic expression of each protein of the complex. However, no evidence for the three-body complex was presented and the role(s) of molecular chaperones in the acquisition of chemoresistance of colon cancer cells was not discussed.

The data presented herein indicates that heat shock proteins can directly help cancer cells to survive the cytotoxic effect of chemotherapeutics. In accordance with this statement recent findings showed that HSPs could empower the evolution of resistance to hormonal therapy in human breast cancer [109], providing support for the notion that the evolutionarily ancient role of heat shock proteins in helping cells to adapt, survive and proliferate is co-opted by cancer cells [81]. One of the mechanisms of adaptive cellular responses under chemotherapeutic selective pressure could be the interaction of molecular chaperones with tumor suppressor p53. We discovered that transient interaction of molecular chaperones with WT p53 is required for transcriptional activity of p53 tumor suppressor protein [76, 77]. Stable interactions of molecular chaperones with p53 were shown to occur when p53 possessed conformational mutations [72, 73, 78]. Nevertheless, we cannot exclude the possibility that due to the stress response induced by chemotherapy, genetically WT p53 could undergo a conformational change by stress conditions or/and by post-translational modifications, allowing the formation of a WT p53-TAp73 complex. It was shown before that phosphorylation of WT p53 at S269 induces mutant conformation of p53 [110]. If such a mechanism really exists *in vivo*, the findings presented in this paper could possess a much more broad impact and may explain the oncogenic activity of elevated MDM2, independent of p53 status.

## MATERIALS AND METHODS

### Cell culture, transfection and treatment

All H1299 cell lines (p53-null, non-small cell lung cancer cells, ATCC CRL-5803^™^) used in this study were maintained in RPMI-1640 medium supplemented with 10% FBS and antibiotics (Sigma-Aldrich). MCF7 (*TP53* ^+/+,^ breast adenocarcinoma ATCC HTB-22^™^) were maintained in Dulbecco’s Modified Eagle’s Medium - high glucose supplemented with 10% FBS, 0.01 mg/ml human recombinant insulin and antibiotics (Sigma-Aldrich). SKBR3 (p53 R175H, breast invasive carcinoma ATCC HTB-30^™^) were maintained in McCoy’s 5a Modified medium supplemented with 10% FBS and antibiotics (Sigma-Aldrich). All cells were grown at 37°C in a 5% CO_2_ humidified incubator. H1299-R175H (H1299c41, a kind gift from G. Blandino) and H1299-R273H cell lines were stably transfected with Ecdysone-inducible Mammalian Expression System (Invitrogen). H1299-R175H-MDM2 cell line was additionally double-transduced with a system of lentiviral vectors to induce MDM2 expression (pLenti CMV/TO Hygro DEST [Addgene #17291] with cloned *MDM2* CDS with 3′ UTR and pLenti CMV TetR Blast [Addgene #17492]; a gift from Eric Campeau [111]). Lentiviruses were produced in 293T human embryonic kidney cells using second-generation packaging system as previously described [112]. Viral supernatants were collected 48 hours post-transfection, concentrated on sucrose cushion and tittered using reverse transcriptase (RT) assay. In order to create the stable constitutive MCF7 and SKBR3 cell line panel, very low passage cells were double-transduced with appropriate lentiviral vectors (modified pLVTH-shp53 described below) at multiplicity of infection MOI = 20 for 24 hours and then selected with 1 µg/ml puromycin.

Cellular transfections with plasmids, SIMP peptides ([88]; LipoPharm) and siRNA HSP40 (Silencer Select^®^ 1/1 mixture of #s7010 and #s223882, Life Technologies/Thermo Fisher Scientific), control siRNA (Silencer^®^ Select Negative Control No. 1 siRNA #4390843, Negative Control No. 2 siRNA #4390846) were performed with Lipofectamine 2000 (Invitrogen) or GenMute (SignaGen^®^ Laboratories) according to manufacturer’s instructions. Cells were treated with Ponasterone A (Invitrogen), Doxycycline (Sigma-Aldrich), Cisplatin (Tocris Bioscience), Camptothecin (Selleck Chemicals), Doxorubicin (Tocris Bioscience), Etoposide (Tocris Bioscience), Taxol (Tocris Bioscience), at the following concentrations: 0.5–3 µM, 50 ng/ml, 10–80 µM, 1–5 µM, 0.25–2.5 µM, 40–80 μM, 0.1–0.5 μM, respectively.

### Plasmids

pCMV plasmids (vector backbone from Clontech) encoding wild type (WT) p53 or p53 mutants (V143A, R175H, G245S, R248Q, R249S, R273H, R282W) were obtained by D. Walerych. pCMV plasmids with p53 mutants R175A, R158L, Y220C, R248W, D281G were obtained by *in vitro* site-directed mutagenesis. pCDNA3.1 plasmids (Invitrogen) encoding HA tagged HSP70 (WT or K71S) were obtained by G.Kudla. The plasmids encoding p63, p73 and MDM2 were pRc-TAp63α, pcDNA3.1-TAp73α and pCMV-MDM2, respectively. pcDNA3.1-HA-TAp73α plasmid was a kind gift from G. Blandino. The plasmid pLVTH-shp53, enabling efficient knockdown of *TP53* gene by the RNAi approach, was described previously [112]. The DNA constructs for the expression of exogenous *TP53* were synthesized commercially (GeneArt Gene Synthesis, Life technologies) and cloned into the pLVTH-shp53 vector at the *MluI and NdeI* sites of the original construct. The designed constructs (puro-2A-FLAG-*TP53*) included puromycin resistance gene, sequences encoding self-cleaving 2A peptide and Flag-tagged *TP53*. The constructs carried either wild type or hot-spot mutants - R175H, R273H, all of which were resistant to the shRNA as a result of introduced silent mutations that did not affect the protein sequence.

### Antibodies

The following antibodies were used for Western Blot and co-immunoprecipitation: p53 (DO-1 and CM-1) and MDM2 (4B2) were a kind gift from B.Vojtesek, p63 (4A4, Santa Cruz Biotechnology), p73 (5B429 mouse monoclonal, Abcam; rabbit polyclonal – gift from B.Vojtesek), HSP70 (SPA-812, Stressgen; 6B3, Cell Signaling), HSP40 (SPA-400, Stressgen), HSP90 (SPA-835, Stressgen), HSC70 (SPA-815, Stressgen), HA (3F10, Roche Molecular Biochemicals; 12CA5, Abcam), PARP (9542S, Cell Signaling), β-actin-HRP (AC-15, Sigma-Aldrich). Secondary antibodies used in Western blot were conjugated with HRP (Calbiochem).

### Co-immunoprecipitation (Co-IP)

H1299 cells were grown to 90% confluence in 6-well or 60 mm plates and transfected with plasmids/peptides/siRNA. Subsequent steps of the assay were performed as previously described [79]. In this study Dynabeads protein A or G (Life technologies) were used and precipitated protein complexes were eluted from beads with Laemmli Sample Buffer resolved in SDS-PAGE system and blotted.

### Two step Co-IP

Cells were grown to 90% confluence in 6-well or 60 mm plates and transfected with plasmids. After 24 h cellular proteins were cross-linked with 1 mM DSP (dithiobis(succinimidyl propionate), Thermo Scientific) in PBS. Reaction was stopped after 30 min by adding Tris-HCl pH 7,5 to the final concentration 20 mM. First co-IP was carried out as described above. Precipitated protein complexes were eluted from beads with 100 mM glicyne pH 2,5 and solution was immediately neutralized by adding Tris-HCl pH 8,0 to the final concentration 150 mM. Eluted proteins were diluted with IP buffer [79] and second Co-IP was performed as described above. Antibodies used for immunoprecipitation were cross-linked to Dynabeads with 20 mM DMP (dimethyl pimelimidate, Sigma-Aldrich) in 200 mM triethanolamine pH 8,2 (Sigma-Aldrich) for 45 min.

### Dot blot analysis

H1299-R175H cells were seeded in 60 mm plates and Ponasterone A (0,5 μM) was added to induce p53 R175H protein. Subsequent steps of the assay were performed as previously described [88].

### Dual luciferase reporter assay

All steps of the assay were carried out as described previously [75, 79].

### Detection of apoptotic cells

Cells were grown to 40–50% confluence in 6-well plates and drugs were added next day. After 24 h (Cisplatin, Camptothecin, Doxorubicin) or 48 h (Etoposide) cells were trypsinized, span down and washed twice with PBS. Washed cells were suspended in 100 μl of Annexin V Binding Buffer (BD Pharmingen) containing 5 μl of Annexin V conjugated with Alexa Fluor^®^647 fluorophore (Biolegend) and 5 μl of 7-Aminoactinomycin D (7-AAD, BD Pharmingen) or GelGreen dye (Biotium) diluted 1:10^4^. After 20 min 400 μl of Annexin V Binding Buffer was added and probes were analysed with flow cytometer (BD FACSCalibur, BD Biosciences) using FL1 (GelGreen dye), FL3 (7-AAD) or FL4 (Alexa Fluor^®^ 647 Annexin V) detectors. Data analysis was carried out with FCS Express 4 Software.

### Real time PCR

H1299 cells were grown to 90% confluence in 12-well plates and transfected with plasmids. After 24 h total RNA was extracted using GeneMATRIX Universal RNA Purification Kit (Eurx). Reverse transcription was performed on 1 µg of total RNA using Eurx reagents (10 ng/μl Random Hexamers, 1 mM dNTPs, 5 mM DTT, 35 U AMV Reverse Transcriptase, 5× Reaction Buffer). TaqMan^®^ Gene Expression Assays and Master Mix (Life Technologies) were used to quantify mRNA levels with Real Time PCR 7900HT apparatus according to manufacturer’s instructions (Life Technologies).

### Monitoring of living cells with xCELLigence system

5 × 10^3^ cells were seeded in each well of E-plate 16 (ACEA Biosciences) and placed in the xCELLigence RTCA DP Instrument (ACEA Biosciences) in 37°C, 5% CO_2_ humidified incubator. After 24 h cisplatin was added and cells propagation was monitored with RTCA Software 2.0 (ACEA Biosciences). For experiments with Doxorubicin dose response, the addition time was moved to 48 hours, and initial seeding was 2.5 × 10^3^ cells. Subsequent data analysis was carried out with the RTCA and PRISM 6 software.

### TCGA data analysis

The TCGA PANCAN12 dataset was downloaded from the UCSC Cancer Genomics Browser [82]. Expression of *MDM2* gene was dichotomized into low/high groups with the median expression, from all the 12 cancer subtypes included in the database, used as the cutoff. Somatic mutations for *TP53* were coded as WT/mut. Complete data for *MDM2* expression, mutations in *TP53,* expression levels and clinical survival was available for 2794 patients ranging the 12 cancer subtypes, which included 745 patients of the TCGA breast cancer cohort and 150 patients of the TCGA lung adenocarcinoma cohort. This analysis was repeated separately for patients for whom the expression of *DNAJB1/HSP40* was lower or higher than median. Note that in this case the *MDM2* gene was dichotomized again with medians calculated in subgroups of patients with lower or higher DNAJB1, respectively. Data snapshots for presented analyses are available at https://github.com/RTCGA/RTCGA.PANCAN12 [113]. All statistical analyses were performed with the R software, version 3.2.2 [114]. Kaplan-Meier curves and log-rank tests are calculated with the package ‘survival’ [115]. The *TP53* copy-number alteration (CNA) data were retrieved from TCGA PANCAN12 and TCGA PANCAN12 BRCA dataset by using http://www.cbioportal.org. The levels of copy-number amplification (CNA) were derived from the copy-number analysis algorithms GISTIC or RAE, and indicate the copy-number level per gene. “-2” is a deep loss, possibly a homozygous deletion, “-1” is a shallow loss (possibly heterozygous deletion), “0” is diploid, “1” indicates a low-level gain, and “2” is a high-level amplification. The classification of breast cancer samples into PAM50 subtypes (Basal, HER2+, LumA, LumB and Normal) was performed using clinical data for TCGA.

## SUPPLEMENTARY MATERIALS FIGURES


