# Supplementary material for: Molecular chaperones in the acquisition of cancer cell chemoresistance with mutated TP53 and MDM2 up-regulation
Source: Oncotarget. 2017 Jun 30;8(47):82123–43. doi: 10.18632/oncotarget.18899 (PMC5669876; doi:10.18632/oncotarget.18899)
Supplement: Supplementary file 1 [file oncotarget-08-82123-s001.pdf]

Molecular chaperones in the acquisition of cancer cell chemoresistance with mutated *TP53* and MDM2 up-regulation

Supplementary Materials

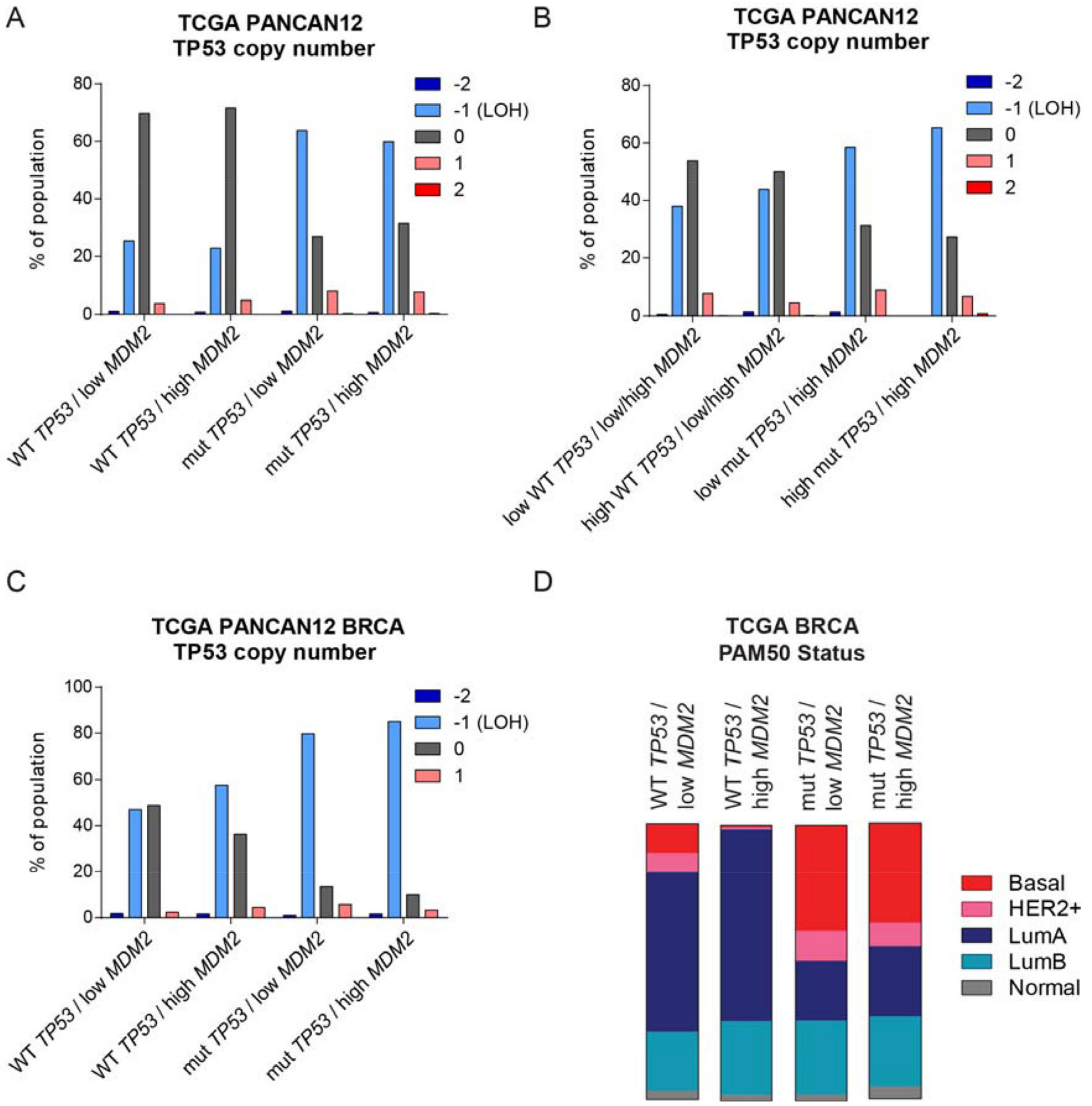

Supplementary Figure 1: TP53 copy number alterations (**A**–**C**). Frequency of homozygous deletions (-2); heterozygous deletions (-1) – loss of heterozygosity (LOH); no change (0); gain (1); high level amplification (2) of *TP53* across the TCGA PANCAN12 (A, B) and TCGA PANCAN12 BRCA (C) patients. (**D**) The association between status of *TP53*/expression of *MDM2* and PAM50 status depicted for TCGA PANCAN12 BRCA patients.

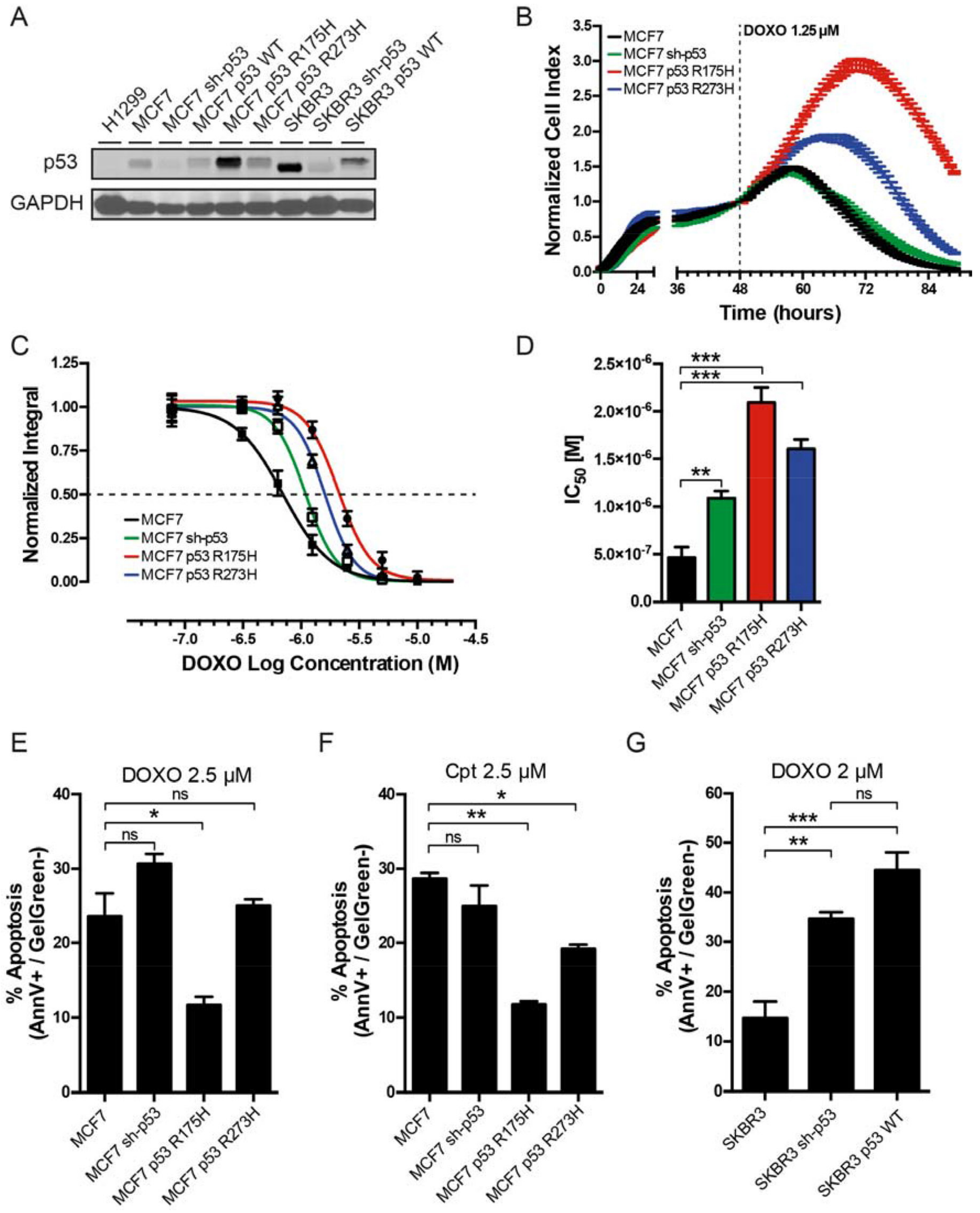

Supplementary Figure 2: The status of p53 in breast cancer cells modulates their resistance to DNA damaging agents. (**A**) Western blot analysis of the generated constitutive cell line panel in comparison to H1299 cell line (p53 null). Immunoblotting was carried out with specific DO-1 antibody. (**B**) The cell lines, stably expressing the indicated p53 mutants, were grown in triplicate in chambers compatible with the xCELLigence RTCA DP instrument. After 48 hours the cells were subjected to Doxorubicin (DOXO). Proliferative index was monitored for 100 h. For clarity, the graph representing the response to one of the seven serial dilutions of the drug is presented. Mean and standard deviation of three repeats are shown. (**C**) Dose response curves for the indicated cell lines were obtained by plotting DOXO concentration – X-axis against the calculated area under the curve (integral from the normalization point - drug addition till the end of the experiment) – Y-axis (**D**) Summary histogram of the calculated IC_{50} values of DOXO for the studied cell lines. Statistical significance (*P* value) was counted for four independent experiments with Anova statistical test. Apoptotic response measurements of the MCF7 stable cell lines, treated with (**E**) Doxorubicin (DOXO) (**F**) Camptothecin (Cpt). (**G**) Apoptotic response measurements of the SKBR3 stable cell lines treated with Doxorubicin (DOXO). The measurement of apoptotic cells was performed with flow cytometry. Bars represent the percentage of cells in early apoptosis (Annexin V positive, GelGreen negative), normalized to non-treated control. Statistical significance (*P* value) was counted for three independent experiments with Anova statistical test. *, **, ***, indicate statistical significance $p < 0.05$, $p < 0.01$, $p < 0.001$, respectively, ns indicates statistical insignificance.

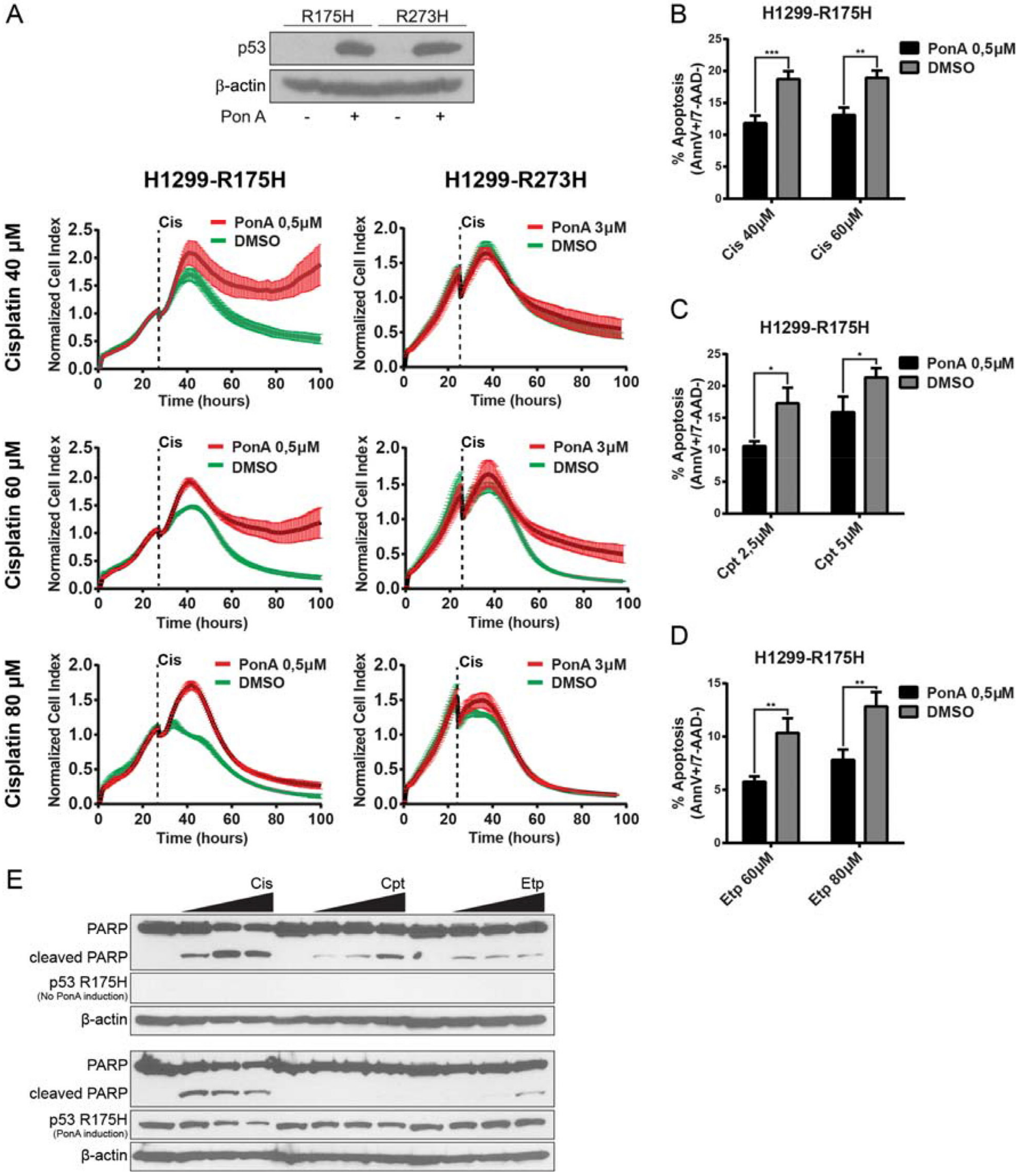

Supplementary Figure 3: H1299 cells expressing structural mutant p53 R175H acquire resistance to DNA damaging agents. (**A**) Stable ecdysone-inducible H1299 cell lines were treated with Ponasterone A (Pon A) for 24 h to induce expression of p53 R175H (0,5 µM PonA) or p53 R273H (3 µM PonA). Immunoblotting was carried out with specific DO-1 antibody. Induced and uninduced cells were grown in triplicate in chambers compatible with the xCELLigence RTCA DP Instrument and three different concentrations of Cisplatin (40, 60, 80 µM) were added at the indicated time points. Proliferative index was monitored for 100 h. Mean and standard deviation of three repeats are shown. Apoptotic response measurements of H1299 cells with induced p53 R175H compared to uninduced (DMSO control), treated with (**B**) Cisplatin/Cis/ (**C**) Camptothecin/Cpt/ and (**D**) Etoposide/Etp/. Cells were treated with the drugs for 24 h (Cis, Cpt) or 48 h (Etp), harvested and stained with Annexin V and 7-AAD. The measurement of apoptotic cells was performed with flow cytometry. Bars represent the percentage of cells in early apoptosis (Annexin V positive, 7-AAD negative), normalized to non-treated control. Statistical significance (*P* value) was counted for three independent experiments with Anova statistical test. *, **, ***, indicate statistical significance $p < 0.05$, $p < 0.01$, $p < 0.001$, respectively. (**E**) H1299-R175H cells were treated with 0,5 µM Ponasterone A (PonA) for 24 h to induce p53 R175H and DNA-damaging drugs were added for 48 h as follows: Cisplatin - 40, 50, 60 µM; Camptothecin - 1, 2, 4 µM; Etoposide - 40, 50, 60 µM. Cleavage of PARP is a marker of caspase 3 - mediated apoptosis.

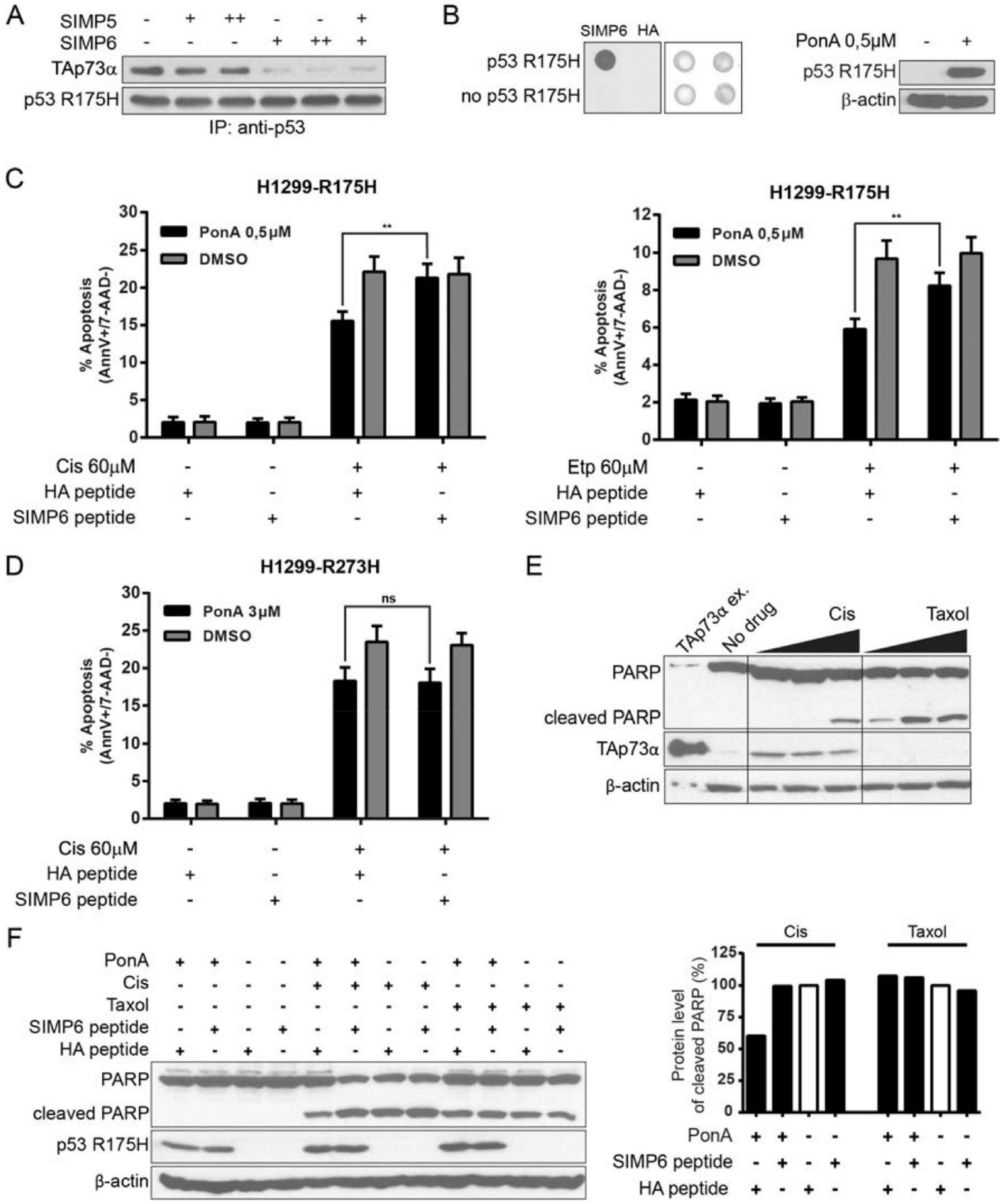

Supplementary Figure 4: Chemoresistance of H1299 cells expressing p53 R175H partly results from inhibition of TAp73-dependent apoptosis. (**A**) SIMP5 and SIMP6 peptides disrupting p53 R175H-TAp73α protein interaction [1] were tested in this study. H1299 cells were transfected with peptides (25–50 μM) and plasmids encoding p53 R175H and TAp73α. 24 h post-transfection cells were lysed, protein complexes were immunoprecipitated with anti-p53 antibody and subjected to Western blot analysis. (**B**) Due to more efficient disruption of p53 R175H-TAp73α complex, the specificity of SIMP6 peptide binding toward p53 R175H protein was verified using Dot blot method. HA peptide showing no affinity for p53 R175H was used as a control. The blot was Ponceau stained prior to Dot blot to verify equal adsorption of the peptides to the membrane. (**C**) Stable ecdysone-inducible p53 R175H H1299 cell line was transfected with SIMP6 peptide or HA control peptide (40 μM). 4 h post-transfection cells were treated with 0,5 μM PonA for 24 h to induce p53 R175H expression, or DMSO (negative control for induction). Afterwards the cells were treated with Cisplatin /Cis/ –24 h or Etoposide /Etp/ –48 h, harvested and stained with Annexin V/7-AAD. Bars represent the percentage of cells in early apoptosis (Annexin V positive, 7-AAD negative). The measurement of apoptotic cells was performed with flow cytometry. Statistical significance (p value) was counted for three independent experiments with Anova statistical test. ** indicates statistical significance $p < 0.01$. (**D**) SIMP6 peptide treatment did not influence apoptotic response of H1299 cells with (or without) p53 R273H induction. Experimental setup was carried out as described in (C). Statistical significance (P value) was counted for three independent experiments with Anova statistical test. ns indicates statistical insignificance. (**E**) Cisplatin induces expression of endogenous TAp73α. H1299 cells were treated with increasing concentrations of Cisplatin (10, 20, 40 μM) and Taxol (0.1, 0.25, 0.5 μM). Induction of apoptotic response was monitored by PARP cleavage. Cell lysate with exogenously expressed TAp73α (TAp73α ex.) was used to define the proper molecular weight of endogenous protein (First lane). (**F**) Cisplatin-induced apoptosis depends on the activity of p73. Stable ecdysone-inducible p53 R175H H1299 cell line was transfected with SIMP6 peptide or HA control peptide (40 μM) and treated with 0,5 μM Ponasterone A (PonA) for 24 h to induce p53 R175H. After treatment with Cisplatin (40 μM) or Taxol (0.25 μM) for 48 h cell lysates were subjected to Western blot analysis to determine the apoptotic response measured by PARP cleavage (left panel). Differences in the level of cleaved PARP were determined by densitometric analysis (right panel). The values obtained from three independent biological experiments were normalized to cleaved PARP of uninduced cells transfected with control peptide treated with chemotherapeutic drug (white bars).

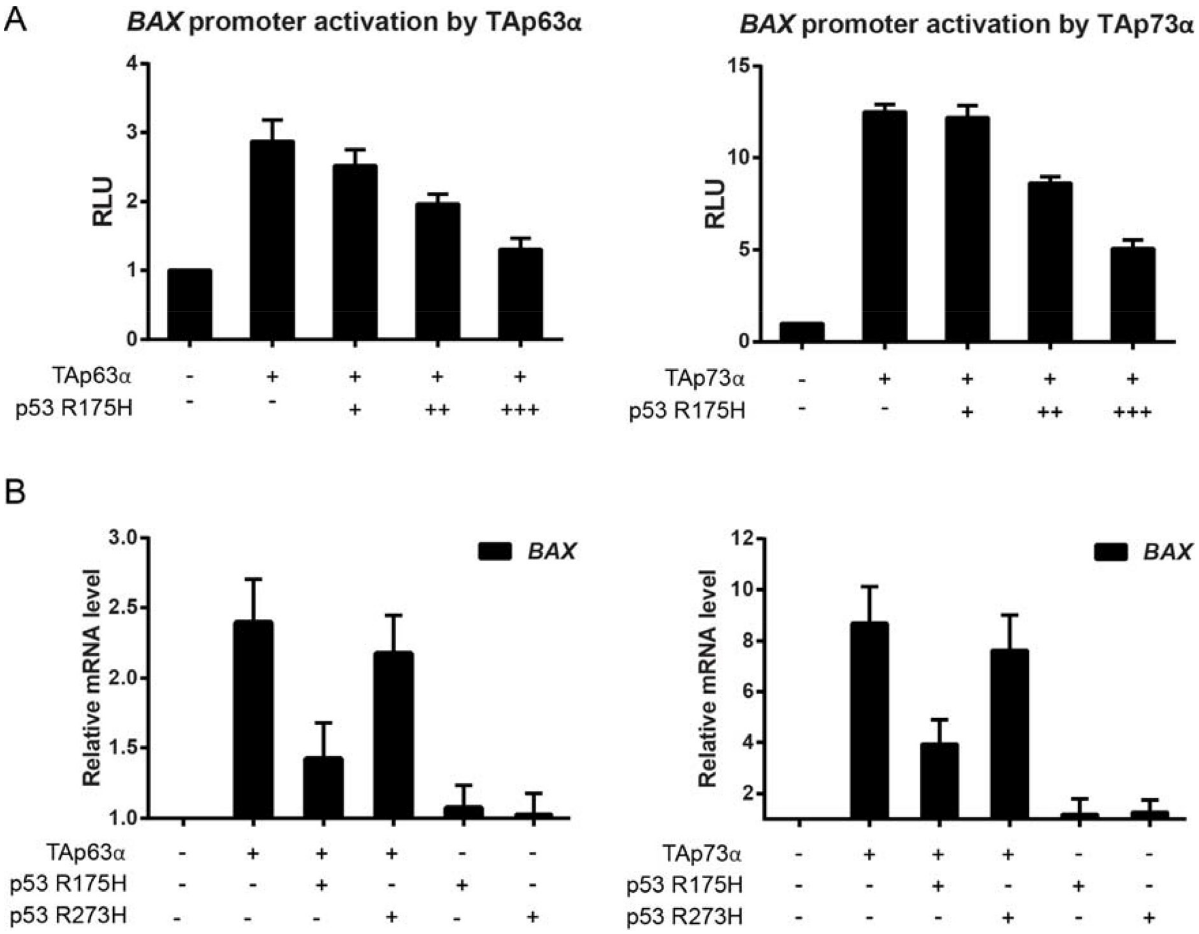

Supplementary Figure 5: p53 mutants with conformational change inhibit transcriptional activity of TAp63α and TAp73α. (**A**) H1299 cells were transfected with plasmids encoding *Firefly luciferase* reporter gene under *BAX* promoter (*BAX*::luc), constitutive *Renilla luciferase* reporter gene, TAp63α or TAp73α isoform and p53 R175H. 24 h post-transfection cells were lysed and chemiluminescence was measured. Transcriptional activity of TAp63α and TAp73α is proportional to the expression of *Firefly luciferase* reporter gene conjugated to the *BAX* promoter. Mean and standard deviation of three independent experiments are shown. (**B**) p53 R175H structural mutant but not p53 R273H contact mutant decreases endogenous *BAX* mRNA levels. Cells were transfected with plasmids encoding TAp63α or TAp73α isoform and p53 R175H or p53 R273H mutant. 24 h post-transfection total RNA was extracted and mRNA level of *BAX* was measured using Real-Time PCR. Mean and standard deviation of three independent experiments are shown.

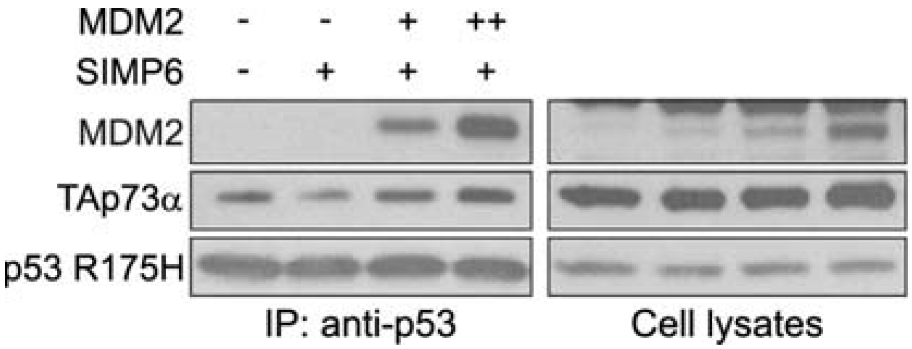

Supplementary Figure 6: p53 R175H-TAp73α complex is resistant to SIMP6 peptide under MDM2 overproduction. H1299 cells were transfected with plasmids encoding p53 R175H, TAp73α, MDM2 and, after 6 h, with 40 µM SIMP6 peptide. 24 h post-transfection cells were lysed and p53 protein was immunoprecipitated with anti-p53 antibody. The immunoprecipitated protein complexes were analyzed by Western blot.

REFERENCES

1. Di Agostino S, Cortese G, Monti O, Dell'Orso S, Sacchi A, Eisenstein M, Citro G, Strano S, Blandino G. The disruption of the protein complex mutantp53/p73 increases selectively the response of tumor cells to anticancer drugs. Cell Cycle. 2008; 7:3440–7. doi: 10.4161/cc.7.21.6995.
